# CellPredX, a computational framework for cross-data type, cross-sample, and cross-protocol cell type annotation through domain adaptation and deep metric learning

**DOI:** 10.1371/journal.pcbi.1013824

**Published:** 2026-01-02

**Authors:** Yan Liu, Yu Xia, He Yan, Long-Chen Shen, Yiheng Zhu, Ji-Peng Qiang, Guo Wei

**Affiliations:** 1 Department of Computer Science, Yangzhou University, Yangzhou, China; 2 College of Information Science and Technology & Artificial Intelligence, Nanjing Forestry University, Nanjing, P. R. China; 3 School of Computer Science and Engineering, Nanjing University of Science and Technology, Nanjing, China; 4 College of Artificial Inteligence, Nanjing Agricultural University, Nanjing, Jiangsu Province, China; 5 School of Life Sciences, Nanjing University, Nanjing, Jiangsu Province, China; Fudan University - Handan Campus: Fudan University, CHINA

## Abstract

Accurate cell type annotation is fundamental to single-cell analysis, yet remains challenging across heterogeneous datasets and modalities. In particular, transferring labels between scRNA-seq and scATAC-seq data poses unique difficulties due to discrepancies in sequencing protocols and feature spaces. Existing methods typically handle only a subset of these challenges, often requiring scenario-specific adjustments and offering limited interpretability. Here, we present CellPredX, a structurally unified but adaptively parameterized, semi-supervised cross-modality framework for label transfer across scRNA-seq, scATAC-seq, and cross-protocol datasets. While maintaining a unified model architecture and optimization strategy, CellPredX allows adaptive tuning of loss-weight hyperparameters to account for the varying degree of similarity or discrepancy between different reference–query dataset pairs. CellPredX integrates domain adaptation and deep metric learning to align heterogeneous embeddings, and introduces a sparse center loss with an attention mechanism to enhance discriminative representations while suppressing noise. Moreover, an integrated interpreter module based on gradient attribution enables biological interpretability by identifying key markers and feature dimensions driving model predictions. Through extensive benchmarking across scRNA to scATAC, scATAC to scATAC, and scRNA to scRNA transfers, CellPredX consistently outperforms state-of-the-art annotation methods in both accuracy and robustness. The interpreter module further reveals biologically meaningful marker patterns that are consistent with known cell hierarchies. Together, these results demonstrate that CellPredX provides an interpretable and scalable solution for cross-modality cell type annotation in single-cell multi-omic integration.

## Author summary

Accurate cell type annotation is crucial for single-cell analysis, yet remains difficult when data come from different modalities or sequencing protocols, such as scRNA-seq and scATAC-seq. Differences in feature space, noise levels, and batch effects often hinder effective label transfer, and many existing methods work only in specific scenarios or lack interpretability. We introduce CellPredX, a unified semi-supervised framework for cell type annotation across scRNA-seq, scATAC-seq, and cross-protocol datasets. CellPredX integrates domain adaptation to align heterogeneous data distributions and deep metric learning to learn discriminative embeddings. A sparse center loss reduces noise and enhances representation quality, while an Integrated Gradients–based interpreter identifies key genes contributing to predictions, improving biological transparency.Across 22 benchmark datasets, CellPredX consistently outperforms state-of-the-art methods in accuracy and robustness. These results show that CellPredX provides an effective, scalable, and interpretable solution for cross-modality cell type annotation in single-cell multi-omics.

## Introduction

Both single-cell RNA sequencing (scRNA-seq) and single-cell Assay for Transposase-Accessible Chromatin sequencing (scATAC-seq) data offer deep insights into transcriptional regulatory networks [1], cell states, and developmental trajectories [2], as well as underlying biological mechanisms, processes, and pathways [3] in cells and tissues. A critical step in analysing scRNA-seq and scATAC-seq data is cell type annotation. For scRNA-seq data [4–9], cell type annotation is typically performed using one of the following two approaches: (i) clustering transcription profiles and assigning cell types based on marker genes that are specific to known cell types [10], or (ii) transferring cell type labels from a well-annotated reference dataset to a query dataset using a label propagation algorithm [5,11–13]. A common approach to cell type annotation using scATAC-seq data involves transferring labels from a well-annotated reference dataset to a query dataset. Depending on the nature of the reference dataset, this process can be performed in two ways: (i) if an annotated scATAC-seq dataset can be used as a reference, the relationship between peaks (i.e., chromatin regions with higher accessibility) and cell types can be learned directly and then used to transfer cell types to the query dataset [14–17]; or (ii) if an annotated scRNA-seq dataset can serve as a reference [18–21], scATAC-seq data can be first transferred into a gene activity matrix (GAM) based on the prior knowledge of regulatory relationship between chromatin accessibility and genes, and model learning and label propagation will then be performed. When using scRNA-seq data as a reference dataset, two scenarios can arise: matched and unmatched cell type annotations. In matched cell type annotations, scRNA-seq and scATAC-seq data originate from the same cells, providing a synchronized view of the transcriptome and chromatin accessibility. This synchronization improves the accuracy and depth of cell type classification by capturing complementary molecular information within the same cellular context. In contrast, unmatched cell type annotation arises when the scRNA-seq and scATAC-seq data do not originate from the same cells, potentially introducing discrepancies in cell type identification and interpretation by variations in cellular conditions and states captured by each sequencing method.

Due to the heterogeneity across reference and query datasets, some significant challenges need to be addressed, such as batch effects induced by distinct protocols or biological differences between scRNA-seq and scATAC-seq. Methods such as scGPT [6], scBERT [7], itClust [22], and scArches [23] apply label propagation within scRNA-seq data, which usually limits their applicability. Although these methods theoretically can annotate cell types by transforming scATAC-seq data into scRNA-seq data, differences in data modality, including variations in gene expression coverage, signal-to-noise ratios, and biological context, still present challenges, complicating the direct application of algorithms designed primarily for scRNA-seq to scATAC-seq data. Methods specifically designed for label propagation within scATAC-seq data, such as EpiAnno [14] and Cellcano [16], can be applied to scRNA-seq data. However, they struggle to sufficiently correct batch effects due to disparities in the feature space. scATAC-seq data typically consist of binary peak accessibility signals, whereas scRNA-seq data contain continuous gene expression values with much higher variability. Batch effects in scRNA-seq are often more pronounced due to differences in sequencing depth, capture efficiency, and technical noise, posing a significant challenge for models designed for peak-based data to fully correct these effects. Conversely, approaches developed for transferring labels from scRNA-seq to scATAC-seq data, including scJoint [18], scNCL [19], and Portal [21], can overcorrect the batch effect when applied to the same data type (i.e., scRNA-seq data or scATAC-seq data), due to substantial modal discrepancies. Moreover, state-of-the-art methods for cell type annotation often use ‘black-box’ models and lack interpretability, further limiting their practical utility.

Inspired by domain adaptation [24] and deep metric learning [25], we propose CellPredX, a unified model for cell type annotation via accurate label transfer across scRNA-seq and scATAC-seq datasets from diverse experimental conditions. CellPredX facilitates label transfer across diverse scenarios, including scRNA-seq datasets from different sequencing protocols, matched or unmatched scRNA-seq and scATAC-seq datasets, and even between scATAC-seq datasets originating from different experimental setups. CellPredX has demonstrated remarkable performance on 22 benchmark datasets, demonstrating its robustness and adaptability in handling complex, multi-modal genomic data for precise cell type annotation. We conducted comprehensive experiments across data types, samples, and sequencing protocols. Additionally, we have developed an interpreter module using the ‘Integrated Gradients’ technique [26] for exploring the underlying mechanisms for CellPredX to make predictions, making CellPredX an interpretable machine-learning framework for cell type annotation.

## Results

### An overview of the CellPredX framework

**Fig 1:**
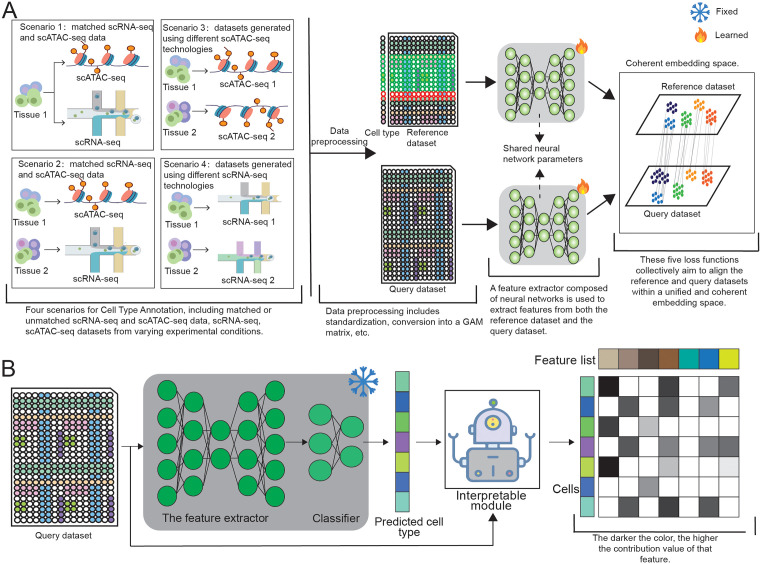
A schematic illustration of the CellPredX framework. **(A)** Four scenarios of cross-modal cell type annotation are illustrated, including matched or unmatched scRNA-seq and scATAC-seq data, as well as datasets generated using different sequencing technologies. Reference and query datasets are preprocessed and passed through a shared feature extractor to obtain aligned embeddings. A combination of five loss functions jointly aligns both datasets within a unified embedding space, enabling robust label transfer across diverse conditions. **(B)** For cell type annotation, the query dataset is fed into a feature extractor and classifier with fixed parameters to obtain predicted cell types. Then, the interpretable module identifies the determinant genes for the final cell type annotation, improving the interpretability of the prediction outcomes.

CellPredX is a semi-supervised framework for label transfer, incorporating principles from domain adaptation [24,27] and deep metric learning [25,28–30] (Fig 1). Domain adaptation [27,31] aligns the distributions of the reference and query datasets to ensure consistency between the training and test datasets. While metric learning refines the embedding space by bringing the reference dataset points closer to their corresponding cells in the query dataset and by simultaneously increasing the separation between distinct cell types. To annotate cell types under different experimental scenarios (i.e., label transfer among datasets sequenced by different techniques), we employed five loss functions to optimize feature extraction and cell type annotation parameters (The ablation experiment is presented in S1 Text and S1 and S2 Figs), including (1) Projection Regularization (PR) Loss [18] that regularizes the entire latent space to ensure a structured and informative embedding landscape; (2) Feature Alignment (FA) Loss [18] that explicitly harmonizes the embeddings between reference and query datasets, thereby aligning the multimodal data within a coherent shared space; (3) Cross-Entropy (CE) Loss [32] that facilitates supervised learning using reference data to enhance the learning of discriminative cell type-specific features and improve model accuracy; (4) Contrastive Loss (CL) [33] that preserves the neighborhood structure of query cells in the raw feature space to maintain the intrinsic topological relationships among cell samples; and (5) Sparse Center Loss (SCL) [34] that utilizes pseudo-labeling information from the query dataset alongside actual labels from the reference dataset to refine the embedding space. We further designed and implemented an interpreter module using the ‘Integrated Gradients’ technique [26] to identify the most influential genes in the model’s predictions, thereby improving its transparency and interpretability.

### Cell type transfer from matched scRNA-seq to scATAC-seq data

**Fig 2:**
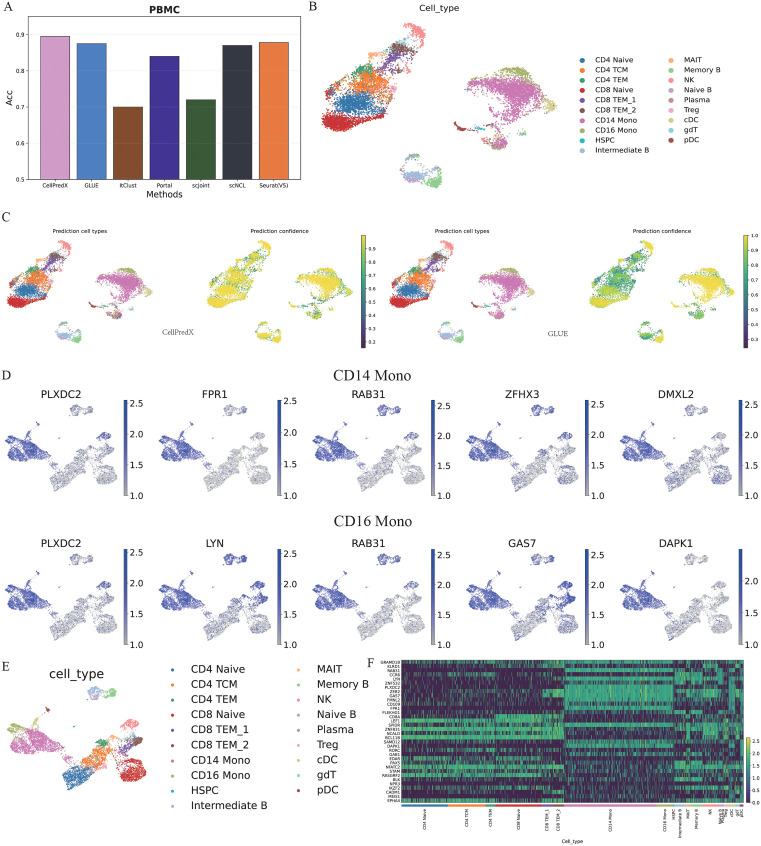
Benchmarking results on matched scRNA-seq (reference) and scATAC-seq (query) PBMC datasets. **(A)** The overall accuracies of CellPredX, GLUE, itClust, Portal, scJoint, scNCL, and Seurat(V5) on the matched PBMC scRNA-seq (PBMC-RNA) and scATAC-seq (PBMC-ATAC) datasets; **(B)** UMAP of PCA embeddings of the PBMC-ATAC dataset with cells colored based on their original cell types; **(C)** UMAP of PCA embeddings of the PBMC-ATAC dataset colored based on the cell-type annotations and prediction confidence by CellPredX and GLUE, respectively; **(D)** Expression profiles of determinant genes for CD14 monocytes and CD16 monocytes; **(E)** UMAP of CellPredX embeddings of the PBMC-ATAC dataset; **(F)** Heatmap for the determinant gene expression profiles of the PBMC-ATAC dataset.

We first evaluated the cell type annotation performance of CellPredX using matched scRNA-seq and scATAC-seq datasets obtained concurrently from the same single cells. On the PBMC dataset (S1 Table), most methods achieved high accuracy (≥0.8) except ItClust, which showed weaker performance likely due to its design for scRNA-seq label transfer rather than cross-modality integration. CellPredX achieved the highest accuracy (0.9), followed by GLUE (0.89) and Seurat(V5) (0.88) (Fig 2A). Consistent with this, CellPredX also showed the best macro-F1 score, indicating robust and balanced performance across cell types (S3 Fig). We then conducted a comparative analysis between CellPredX and GLUE in terms of the confidence levels of their predictions. CellPredX demonstrated significantly higher confidence levels for the most accurately predicted cell types, including CD4 TCM (central memory) cells (Figs 2B, 2C and S4). These results highlight CellPredX’s superior prediction accuracy and prediction confidence, underscoring its reliability and effectiveness as a robust tool for cell type prediction.

An interpreter module was integrated into CellPredX to identify the specific genes based on which the predictions were made (i.e., determinant genes). Figs 2D, S5, and S6 illustrate the expression levels of the five most frequently identified determinant genes (refer to the “Obtaining determinant features for each cell type” section) used to predict each cell type. For example, DAPK1 gene expression was markedly lower in several cell types compared to CD14 and CD16 monocytes, which are associated with inflammatory responses [35]. Consistently, Li et al. demonstrated that siRNA-mediated DAPK1 knockdown in human monocytic THP-1 cells significantly reduced IL-17-induced IL-8 production upon TNF-α and IL-1β stimulation [36], suggesting that DAPK1 may contribute to the inflammatory activities characteristic of CD14⁺ and CD16⁺ monocytes. A heatmap of the determinant genes from the PBMC-ATAC dataset also reveals distinct gene expression patterns across different cell types (Fig 2F), which are not readily distinguishable by visual inspection alone (e.g., CD4 TCM cells and CD4 TEM cells). Despite these different gene expression patterns, CellPredX still accurately separated these two cell clusters. This is because CellPredX predicts cell types based on a nonlinear joint latent representation learned from both gene expression and chromatin accessibility features through domain adaptation and deep metric learning. This learned representation enables a more accurate and nuanced characterization of cell identities by integrating multimodal regulatory information into a unified embedding space. In addition, we introduced a new evaluation metric, Key Gene Hit Rate (KGHR), to further assess the model’s ability to identify key determinant genes (S7A Fig). As shown, CellPredX achieved consistently high KGHR values across most cell types (up to 0.9), indicating its strong capability in capturing representative gene features. These results further demonstrate the superior performance of our method in key gene identification and biological interpretability.

### Cell type transfer from unmatched scRNA-seq to scATAC-seq datasets

**Fig 3:**
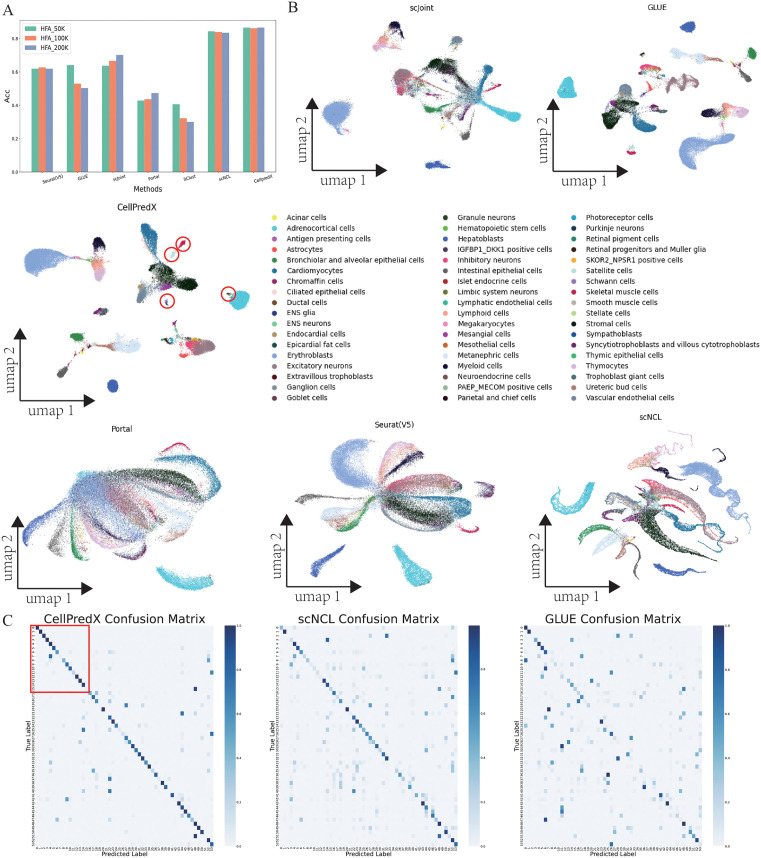
Benchmarking results label transfer between unmatched scRNA-seq and scATAC-seq datasets. **(A)** Overall accuracy of CellPredX, GLUE, itClust, Portal, scJoint, scNCL and Seurat(V5) on HFA_50k, HFA_100k, and HFA_200k dataset; **(B)** UMAP plots of embeddings on the HFA_100k-ATAC dataset by scJoint, GLUE, CellPredX, Portal, Seurat(V5), and scNCL; **(C)** Confusion matrices of the true cell types vs the predicted cell types on the HFA_100K-ATAC dataset.

Compared to matched scRNA-seq and scATAC-seq datasets that are expensive to generate, using unmatched scRNA-seq to scATAC-seq for cell type transfer is more practical. Here, we evaluated the performance of CellPredX in transferring cell type information from unmatched scRNA-seq (reference) to scATAC-seq (query) on three datasets extracted from Human Cell Atlas [37], including HFA_50K, HFA_100K, and HFA_200K (S1 Table). CellPredX consistently achieved the highest performance, with an average accuracy of approximately 0.86 (Fig 3A) and a macro-F1 score around 0.60 (S8 Fig). In contrast, Seurat(V5) and GLUE showed limited accuracy (about 0.62 and 0.54) and lower F1 scores (<0.38), indicating their reduced robustness on large-scale heterogeneous datasets. This performance gap likely stems from fundamental differences in how these methods handle biological variability. Seurat (V5) and GLUE rely primarily on feature-level or manifold-level alignment, assuming that shared gene anchors or co-accessible peaks are sufficient to align modalities. However, when biological heterogeneity, such as cell-type specific chromatin accessibility or transcriptional regulation, dominates, this assumption no longer holds, leading to incomplete integration and degraded performance. In contrast, CellPredX employs a domain adaptation module combined with deep metric learning, enabling adaptive alignment of embeddings even when gene-activity relationships differ substantially across modalities. UMAP plots on the HFA_100K-ATAC dataset revealed that CellPredX preserved distinct clusters in the embedding space, especially for small clusters of specific cell types, such as astrocytes and enteric nervous system (ENS) glia cells (Fig 3B). Furthermore, prediction results by CellPredX demonstrated shorter distances between the cells of the same type compared to scNCL, which achieved the second-highest accuracy (Fig 3C). These results show that CellPredX can maintain a compact representation of cell types in the embedding space to enhance cell type differentiation, thereby predicting cell types more accurately. A detailed examination of the performance metrics for each cell type (Figs 3C and S9) shows that CellPredX achieved high accuracy in predicting both major and minor cell types using scRNA-seq data. In order to facilitate observation, we converted cell types into numerical labels corresponding to the sequences of cell types (Fig 3B). Notably, CellPredX allowed the accurate identification of rare cell types (e.g., acinar and adrenocortical cells as well as astrocytes) within the reference dataset, which may relate to CellPredX’s ability to identify highly associated genes with these cell types (S10 Fig).

### Label transfer between scATAC-seq datasets from different experimental conditions

**Fig 4:**
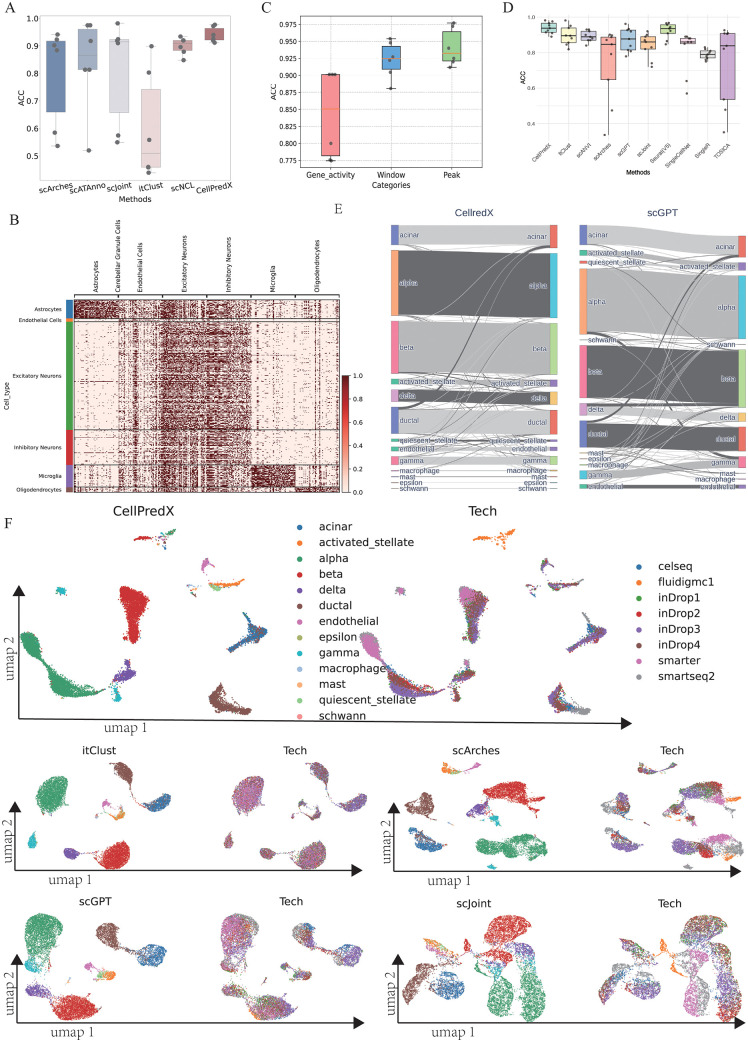
Benchmarking different methods for cell type transfer. **(A)** Overall accuracy for scArches, scATAnno, scJoint, itClust, scNCL, and CellPredX across three mouse brain datasets. Each box summarizes six data points corresponding to all pairwise label transfer directions (i.e., each dataset used once as reference and once as query). **(B)** Peaks that the CellPredX interpreter identified as major contributors to predictions in the mouse brain dataset (10x Genomics); **(C)** The accuracy of CellPredX when using different input representations of scATAC-seq data. It highlights how the choice of input format affects model performance. **(D)** Overall accuracy of CellPredX, itClust, scArches, scGPT, scANVI, SingleCellNet, SingleR, Seurat(V5) and TOSICA on nine human pancreas datasets; **(E)** River plots illustrating the predicted cell types by CellPredX and scGPT and their relationships to the actual cell types on the human pancreas datasets except the reference datasets (i.e., sequenced by celseq2; **(F)** UMAP showing embeddings of the query dataset generated by CellPredX, itClust, scArches, scGPT, and scJoint. Note: “Tech” refers to different sequencing protocols.

In this section, we used three publicly available mouse brain scATAC-seq datasets (S1 Table), with each dataset serving alternately as the reference and query dataset. Figs 4A and S11A illustrate that CellPredX again achieved the highest accuracy and macro-F1 score among the benchmarked methods. It is also worth noticing that CellPredX achieved more accurate cell type transfer across the different scATAC-seq datasets than scATAnno, which was specifically designed for label transfer between scATAC-seq datasets. Subsequently, we plotted heatmaps to analyze the opening intensity of chromatin, revealing that CellPredX can unravel clear patterns for distinct cell types in each dataset, particularly for microglia (Figs 4B and S12). We then transformed the three mouse brain datasets into GAMs and window forms [38] for cell type transfer. The results (Figs 4C and S11B) indicate that using peaks as inputs yielded significantly higher accuracy than gene transcription. The analysis indicated that the accuracy was notably higher when peak features were used compared to when window form inputs were employed. These findings suggest that utilizing the GAM, which aggregates peak signals at the gene level based on existing knowledge of regulatory associations, might lead to a loss of information. Specifically, GAM might not capture cell type-specific chromatin accessibility patterns effectively, especially in scenarios where peaks regulate genes over long distances or involve multiple regulatory elements that influence gene activity in complex, non-linear ways. This can result in a less precise understanding of the chromatin landscape associated with different cell types, potentially overlooking subtle yet crucial regulatory nuances.

### Cell type transfer using scRNA-seq datasets from different sequencing protocols

The challenge in transferring labels between scRNA-seq datasets arises from variations introduced by different sequencing methods/protocols. In this section, we used nine human pancreas datasets sequenced by nine techniques to critically evaluate CellPredX’s performance in predicting cell types across scRNA-seq datasets (S1 Table). Specifically, for each experiment, one dataset sequenced by a particular technique was designated as a reference, while datasets produced using the other eight sequencing technologies served as query datasets. To ensure that the accuracy represents the prediction performance of shared cell types between the query and reference datasets, cells not present in the reference dataset were removed from the query dataset. According to Figs 4D and S13, CellPredX achieved the highest average accuracy and macro-F1 score was more stable than itClust, scArches, scGPT, Seurat(V5), and TOSICA. Using the ‘celseq2’ dataset as reference and other sequence datasets as the queries, CellPredX achieved an accurate classification of cell types (both abundant and minor) represented in the scRNA-seq datasets (Figs 4E, S14, and S15). UMAP plots (Figs 4F and S16) further revealed that CellPredX maintained a tight clustering of individual cell types within the embedding space, allowing accurate cell type differentiation.

### CellPredX can reliably detect novel cell types

**Fig 5:**
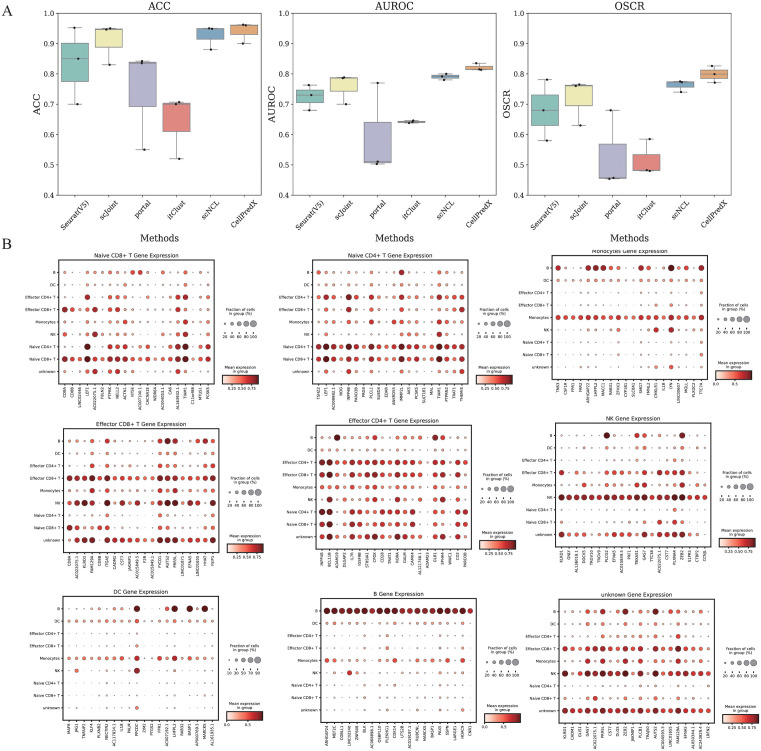
Benchmarking results for the annotation of common and novel cell types. **(A)** Overall accuracy, AUROC, and OSCR of CellPredX, itClust, Portal, scJoint, scNCL, and Seurat(V5) in the novel cell type detection scenario; **(B)** Dotplots of determinant genes of different cell types.

In cell type annotation, the reference and query datasets may not have identical cell types. Therefore, cell type annotation methods should not only reliably identify existing cell types but also be able to infer novel cell types. Since the label transfer across data types (i.e., scRNA-seq and scATAC-seq data) is particularly challenging, in this section, we evaluated the performance of CellPredX in identifying new cell types using the CITE-ASAP, PBMC, and HFA_50K datasets. The CITE-ASAP dataset includes 4,502 CITE-seq and 4,644 ASAP-seq cells, sharing seven common cell types, while dendritic cells (DCs) are unique to the ASAP-seq data. For the PBMC dataset, we excluded CD8 TEM_1 and CD8 TEM_2 from the reference data. Similarly, for the HFA_50K dataset, Adrenocortical cells were removed from the reference dataset. Six methods, including CellPredX, Seurat(V5), scJoint, Portal, Concerto, and itClust, were benchmarked for label transfer on the three datasets. GLUE was excluded from this comparison as it is not able to handle different types of data. The results demonstrated that CellPredX outperformed other methods in ACC, AUROC, and OSCR (Fig 5A) for cell type annotation and can identify novel cell types within data. CellPredX was able to identify determinant genes for each cell type based on their activity (Fig 5B). For instance, KEF1 and TIAM1 were highly transcribed and represented in naive CD8+ T and CD4+ T cells, respectively, whereas CD8A (with moderate/low transcription) represented naive CD4+ T cells. This ability to discern subtle variation in transcription allows CellPredX to accurately identify and distinguish closely related cell types. This gene-level resolution not only supports accurate annotation of known cell types but also empowers CellPredX to recognize potentially novel or previously unannotated populations. On the other hand, for the CITE-ASAP dataset, CellPredX achieved consistently high KGHR values across most cell types (mostly above 0.6, as shown in S7B Fig), indicating its strong ability to capture representative marker genes. These findings further confirm the superior performance of our method in identifying key genes and enhancing biological interpretability.

### Runtime and scalability analysis

**Runtime and Scalability Analysis were provided in** S2 Text and S17 Fig.

## Discussion and conclusion

In this study, we introduced CellPredX, a unified framework for cell type annotation that integrates scRNA seq and scATAC seq data. CellPredX was rigorously validated through multiple experiments for its ability to handle various label transfer scenarios, including transferring annotations between scRNA seq datasets generated using different protocols, between matched scRNA seq and scATAC seq datasets, and among scATAC seq datasets derived from different experimental conditions. The consistent performance of CellPredX across these diverse settings highlights its robustness and effectiveness in managing complex multimodal genomic data for accurate cell type annotation. A significant feature of CellPredX is its built in interpretability module, which enhances users’ understanding of the model’s internal mechanisms. This functionality is particularly useful for identifying potential marker genes and improving the biological transparency of the annotation process.

In cross modality experiments (e.g., scRNA to scATAC), CellPredX relies on converting ATAC peak signals into a GAM to align the feature space between RNA and ATAC modalities. This strategy follows the standard practice in multimodal integration but inevitably introduces a limitation. As demonstrated in our benchmarking analyses, GAM based representations tend to underperform compared with peak level features in ATAC to ATAC transfers, indicating that part of the chromatin accessibility information is lost during conversion. This trade off is inherent to the GAM representation rather than a flaw of CellPredX. Accordingly, we explicitly acknowledge that the cross-modality results reported here are constrained by this lossy transformation, and future work will explore alternative designs that jointly utilize peak level and gene level information to better preserve regulatory signals across modalities.

Despite the overall success of CellPredX, there remain areas for further improvement. (1) CellPredX relies heavily on the quality of the reference dataset, which can significantly influence model training and prediction performance. To enhance flexibility, enabling CellPredX with a zero-shot capability (allowing annotation without a reference) could greatly improve its applicability. (2) The current design primarily leverages data driven learning of cell type specific features, ensuring strong correspondence between model outputs and genomic signals. However, it does not yet incorporate expert biological knowledge, such as prior understanding of lineage trajectories or functional annotations, which could further enhance classification accuracy in complex or ambiguous cases. (3) Furthermore, CellPredX could be extended to handle additional multimodal assays such as Paired tag (RNA + histone modifications) and unpaired CUT&Tag scRNA datasets. The modular design of the feature extractor and interpreter provides the flexibility to adapt to other data modalities. In particular, the interpreter module could be modified to identify determinant chromatin marks in addition to gene level features, thereby extending the interpretability of CellPredX to epigenetic regulation. This represents an exciting direction for future development.

Overall, CellPredX represents a robust and interpretable solution for unified cell type annotation across diverse single cell modalities, and we believe it lays a foundation for future developments toward more biologically informed and reference independent models.

## Materials and methods

### Dataset collection and processing

We trained CellPredX and assessed its ability in annotating cell types using multiple datasets, including human Peripheral Blood Mononuclear data (PBMC) [19], human pancreas data [38], human fetal atlas data [37,39], mouse brain data [38], and stimulation data (i.e., CITE-ASAP dataset) [40]. To evaluate the robustness of our analysis across datasets of different sizes, we performed subsampling on the HFA dataset to create subsets of two experimental protocols with different numbers of cells: 20,000 cells for scRNA-seq and 30,000 cells for scATAC-seq, 40,000 cells for scRNA-seq and 60,000 cells for scATAC-seq, and 80,000 cells for scRNA-seq and 120,000 cells for scATAC-seq. These subsets are then referred to as HFA-subset-50k, HFA-subset-100k, HFA-subset-200k, respectively. In the case of the CITE-ASAP dataset, the log-normalized GEM/GAM matrix and log-normalized ADTs matrix were concatenated. Detailed statistical information regarding the benchmark datasets can be found in S1 Table.

In this study, the cell types identified in the reference do not align with those in the query dataset. Cell types that are present in both reference and query dataset are termed “shared cell types”, whereas those unique to the query dataset are referred to as “new cell types”. The primary objective of cell type annotation extends beyond merely identifying shared cell types; it also encompasses the discovery of new cell types. When transferring labels from scRNA-seq to scATAC-seq data, Signac [41] should be used to transform the scATAC-seq to GAM matrix, and the gene transcription matrix of the scRNA-seq data that shares the feature list with GAM should be identified as the input for CellPredX. While for cell type transfer within scRNA-seq or scATAC-seq, it is important to identify a common set of genes or peaks.

### The computational flow in CellPredX

Let us write $D^{r}$ and $D^{q}$ as the reference and query dataset, respectively. During the training process, after log-normalization on both $D^{r}$ and $D^{q}$, a mini-batch $B^{r}$ and $B^{q}$ from $D^{r}$ and $D^{q}$ is fed into the feature extractor and the classifier network of CellPredX. The feature extractor *f* projects the cells from both $D^{r}$ and $D^{q}$ into a shared embedding space with the dimensionality of m. Subsequently, the classifier network *c* processes these embeddings and outputs a *K*-class probability vector *p*, where K is the number of cell types in $D^{r}$. The predicted class e is determined by $e=\text{m}ax(p_{k})$, where $p_{k}$ is the predicted probability for class k. If e exceeds a specified threshold (This study is set to 0.95), the cell type corresponding to the maximum probability is assigned to the cell; otherwise, the cell is labeled as “Unknown”. In the prediction phase, the parameters of the feature extractor f and the network classifier c need to be fixed, and then only the query dataset is input to obtain the final annotated results.

### Optimization objectives for CellPredX

At each training step, we randomly select the same number of mini-batches $B^{r}$ and $B^{q}$ from $D^{r}$ and $D^{q}$. Similar to scNCL [19], we employ a PR loss to enforce the extraction of low-dimensional and orthogonal features during the projection of each mini-batch into a shared embedding space. The PR loss is defined as:


$$\begin{array}{c} \begin{array}{c} L_{PR}={\left(\frac{\text{1}}{\left|B^{q}\right|}\sum_{b\in B^{q}}\sum_{j=\text{1}}^{d}\left|{{\text{𝒹}}_{b}(j)}^{q}-{\overset{―}{\text{𝒹}}(j)}^{q}\right|\right)}^{-\text{1}}\\ +\frac{\text{1}}{d^{\text{2}}}\sum_{i\neq j}\left|A_{ij}^{q}\right|+\frac{\text{1}}{d}\sum_{j=\text{1}}^{d}\left|{\overset{―}{\text{𝒹}}(j)}^{q}\right|+\frac{\text{1}}{d^{\text{2}}}\sum_{i\neq j}\left|A_{ij}^{r}\right|+\frac{\text{1}}{d}\sum_{j=\text{1}}^{d}\left|{\overset{―}{\text{𝒹}}(j)}^{r}\right|, \end{array} \end{array}$$ (1)

where ${{\text{𝒹}}_{b}(j)}^{q}$ denotes the *j*-th dimension of the embedding of cell b, ${\overset{―}{\text{𝒹}}}^{q}=\frac{\text{1}}{\left|B^{q}\right|}\sum_{i\in B^{q}}{\text{𝒹}}_{i}$ and ${\overset{―}{d}}^{r}=\frac{\text{1}}{\left|B^{r}\right|}\sum_{i\in B^{r}}d_{i}$, and $A_{ij}^{q}$ and $A_{ij}^{r}$ denotes the feature correlation of batch $B^{q}$ and $B^{r}$. To learn cell type-specific features, the CE loss is used to guide the learning of networks:


$$\begin{array}{c} L_{CE}=-\frac{\text{1}}{\left|B^{r}\right|}\sum_{b\in B^{r}}\sum_{k=\text{1}}^{K}\text{1}\left(y_{b}^{r}=k\right)\cdot \text{log}\left(p_{b}(k)\right), \end{array}$$ (2)

where $\left|B^{r}\right|$ denotes the number of samples in $B^{r}$, $y_{b}^{r}$ is the true cell type of cell b and $p_{b}(k)$ denotes the probability that the cell *b* belongs to cell type *k*.

In this study, we employed the CL function to maintain the integrity of the neighborhood graph constructed from the raw query dataset. This approach ensures that the spatial relationships among data points in the graph are preserved, thereby enhancing the model’s ability to accurately reflect the underlying structure of the dataset. The CL can be calculated as:


$$\begin{array}{c} L_{CL}=-log\frac{\text{exp}(\frac{sim\left(x,x^{+}\right)}{\tau })}{\text{exp}(\frac{sim\left(x,x^{+}\right)}{\tau })+\sum_{i=\text{1}}^{\left|B^{q}\right|}\text{exp}(\frac{sim\left(x,x_{i}^{-}\right)}{\tau })}, \end{array}$$ (3)

where sim(x,y) denotes the cosine similarity between *x* and *y*, and τ represents a temperature parameter used to adjust the smoothness of the probability distribution, $x^{+}$ and $x^{-}$ are the positive and negative sample of x.

In CellPredX, to facilitate alignment between the reference dataset ($B^{r}$) and the query dataset ($B^{q}$) within the embedding space, we employed two distinct loss functions: FA loss [18] and SC loss [34]. The FA loss is specifically designed to reduce the distance between corresponding pairs of cells between datasets $B^{r}$ and $B^{q}$, thereby effectively drawing them closer in the embedding space. CellPredX first calculates the cosine similarity for every pair of cells between $B^{r}$ and $B^{q}$ based on their embeddings. High similarity scores between cell pairs suggest a potential match in cell type, indicating that these pairs should be aligned more closely. For each query cell $b\in F_{q}$, we find its most similar reference cell i in $B^{r}$ by maximizing $\text{cos}(d^{q},d^{r})$, and define the FA loss as:


$$\begin{array}{c} L_{FA}\left(B^{q}\right)=-\frac{\text{1}}{\left|F_{P}\right|}\sum_{b\in F_{P}}\text{cos}\left(d_{b}^{q},d_{i}^{r}\right), \end{array}$$ (5)

where $F_{P}$ is the top *p* fraction of cells from $B^{q}$, and i is the index of the reference cell in $B^{r}\;$that achieves the maximum cosine similarity with cell *b*.

Compared to conventional center loss methods [42], we hypothesize that only a subset of dimensions in a cell embedding are truly informative for distinguishing between specific cell types. Our goal is to identify and leverage only these task-relevant embedding components while ignoring irrelevant ones. To this end, we propose a novel loss function, which we term SC loss, that explicitly promotes sparsity by suppressing non-discriminative features during the computation of class centers:


$$\begin{array}{c} L_{SC}=\frac{\text{1}}{\left|B\right|}\sum_{i=\text{1}}^{B}\sum_{j=\text{1}}^{d}a_{ij}⨀{\left\|d_{ij}-c_{y_{i}j}\right\|}^{\text{2}}, \end{array}$$ (6)

where $B=B^{r}\cup B^{q}$, $a_{ij}$ is a binary or continuous weight associated with each feature in the embedding, ⨀ indicates element-wise multiplication, $d_{ij}\;$refers to the *j*-th component of the *i*-th cell embedding, and $c_{y_{i}j}$ denotes the *j*-th component of the center of the class to which the *i*-th cell belongs. A center of a class is typically the mean of all embeddings in that class and is adjusted dynamically during training.

Overall, the training loss function is defined as:


$$\begin{array}{c} L=L_{CE}+\text{0}.\text{1}L_{PR}+{\alpha L}_{CL}+{\beta L}_{FA}+\gamma L_{SC}, \end{array}$$ (7)

where α, β and γ are the balance parameter of different loss terms.

### Attention network

We developed an auxiliary attention network integrated with the feature extractor to dynamically determine the weight vector $a_{i}\in R^{m}$ for each input, where *m* is the dimensionality of the cell embedding. These weights are used in the SC loss to modulate the contribution of each embedding component during training. Specifically, this approach aims to adaptively estimate the weights required for the sparse center loss, tailoring them to the specific demands of the task and the characteristics of the input data. Ideally, these weights are computed by a neural network to ensure responsiveness to data variability. In this regard, we introduced an attention network, denoted as *A*, to adaptively generate an attention weight vector, which specifically regulates the contribution of each cell embedding $x_{i}$ across the *j*-th dimension as outlined in Eq. 6, thereby enhancing the model’s ability to focus on relevant features for improved performance. The attention network *A* comprises two principal components: (1) a context encoder unit (CE-Unit) with a linear layer of *m* dimension, a BatchNorm layer and a Tanh layer to process the input data to generate a latent representation, which captures the essential information needed for subsequent tasks [34]; and (2) a multi-head binary classification module to take the latent representation (i.e., d→e; generated by the CE-Unit) as input. The classification module employs a multi-head mechanism to estimate the attention weights. Each ‘head’ in this module focuses on feature vectors of the input, thereby allowing for a nuanced interpretation and utilization of the latent information. Then, the inclusion and exclusion scores ($p_{ij_{in}}$ and $p_{ij_{ex}}$) for the *j*-th dimension in $d_{i}$ are calculated as follows:


$$p_{ij_{in}}=A_{j_{in}}^{T}e_{i}+b_{j_{in}},$$ (8)

$$\begin{array}{c} p_{ij_{ex}}=A_{j_{ex}}^{T}e_{i}+b_{j_{ex}}, \end{array}$$ (9)

where $A_{j}\in R^{\text{2}*d}$ and $b_{j}\in R^{\text{2}}$ are the learnable weights and biases of each classification. Finally, the corresponding attention weight $a_{ij}$ is calculated as follows:


$$\begin{array}{c} a_{ij}=\frac{\text{exp}\left(p_{ij_{in}}\right)}{\text{exp}\left(p_{ij_{in}}\right)+\text{exp}\left(p_{ij_{ex}}\right)}. \end{array}$$ (10)

In this study, both the query and reference datasets were processed through the attention network. The reference dataset was used with its original cell type labels, whereas pseudo labels were assigned to the query dataset to enable centroid estimation for each cell type. These pseudo labels were derived from the classifier’s predicted cell types during training and were iteratively refined as the model parameters were updated. The refined pseudo labels were subsequently used in the computation of the SC loss to enhance feature alignment between the reference and query embeddings.

### Obtaining determinant features for each cell type

We utilized the Integrated Gradients method embedded in Captum [26] to calculate the contribution score of each feature to query cell type annotation. For each cell, we identified the top 100 features as determinant features sorted in descending order based on their contribution scores. Then, for each cell type, we calculated the frequency of occurrence of each feature within the determinant features of the cells annotated as that cell type and selected the top 50 features with the highest occurrence frequencies as the final determinant features for each cell type.

### Training details

To train CellPredX, we set the batch size to 512, the embedding dimensionality m to 64, e to 32, and the initial learning rate to 0.001. Principal component analysis (PCA) was utilized to reduce the dimensionality of the original query dataset, resulting in 50 principal components retained. Subsequently, *k*-nearest neighbor (KNN) was employed to identify each query cell’s neighbors and construct the comparison loss. The hyperparameters α, β and γ, along with the temperature coefficient τ, were adjusted based on the specific experimental scenarios, considering differences in data modalities and sequencing techniques that require varying degrees of alignment strength. For matched scRNA-seq and scATAC-seq data, α, β, γ, and τ were set to 0.06, 0.05, 0.01, and 0.04, respectively. While for unmatched scRNA-seq and scATAC-seq data, these parameters were adjusted to 1.2, 0.05, 0.01, and 0.8, respectively. For label propagation between scRNA-seq datasets, α, β, γ, and τ were set to 0.1, 0, 0.1, and 0.8, respectively. For label propagation between scATAC-seq datasets, the parameters were set to 0.1, 0, 0.001, and 0.8, respectively. To determine the early stopping criteria for scATAC-seq and scRNA-seq data, we established a maximum iteration limit of 3000, given the substantial modality differences between these data types. Additionally, training is halted if the sparse loss remains unchanged over 20 consecutive rounds. For label propagation within the same data type (i.e., scATAC-seq or scRNA-seq), we set a maximum of 200 iterations. We have provided “Sensitivity Analysis of CellPredX under Different Experimental Settings” in S3 Text and S18 Fig.

### Benchmarking CellPredX against state-of-the-art cell type annotation methods

We benchmarked CellPredX with state-of-the-art cell type annotation approaches in three experimental scenarios, including cell type transfer (i) from scRNA to scATAC-seq data, (ii) between scATAC -seq datasets, (iii) between scATAC-seq datasets, and (iv) between scRNA-seq datasets. For cell type transfer from scRNA-seq to scATAC-seq data, we benchmarked CellPredX with GLUE [20], itClust [22], Portal [21], scJoint [18], scNCL [19], and Seurat(V5) [43]. itClust was specifically designed for label transfer between scRNA-seq datasets, whereas GLUE is a computational framework designed to integrate single-cell multi-omics data by learning a unified latent representation across different modalities, such as gene expression, chromatin accessibility. Portal, scJoint, scNCL and Seurat (V5) were tailored to transfer labels from scRNA-seq to scATAC-seq data. For label transfer between scATAC-seq datasets, we compared CellPredX with scArches [23], scATAnno [14], and scJoint. For label transfer between scRNA-seq datasets, we evaluated the performance of CellPredX against scArches, scGPT [6], scANVI [12], SingleCellNet [44], SingleR [45], and TOSICA [11]. scGPT leverages large-scale pre-trained transformer models that are initially trained on extensive single-cell transcriptomic datasets to capture generalizable gene expression patterns. These pre-trained models enable downstream tasks such as cell type annotation with minimal task-specific fine-tuning. In contrast, TOSICA utilizes a multi-head self-attention mechanism specifically designed for direct cell type classification in scRNA-seq datasets. Detailed information on the parameter configurations for these methods is presented in S4 Text.

### Evaluation metrics

The detailed evaluation metrics, including Accuracy (ACC), and F1-score, are provided in S5 Text.

### Declaration of generative AI and AI-assisted technologies in manuscript preparation

The authors employed ChatGPT 4.0 to enhance the English and correct the grammatical errors. The authors subsequently reviewed and edited the content and take full responsibility for the content in the publication.

## Supporting information

S1 Fig Ablation study on HFA_50k and PBMC datasets. (TIF)

S2 Fig UMAP visualization of CITE-seq and ASP-seq embeddings before and after applying the FA loss. **(A)** Raw embeddings show strong modality-driven separation. **(B)** Embeddings trained without FA loss (“No FA loss”) show partial alignment but still retain modality bias. **(C)** Embeddings trained with all loss components (“All loss”) achieve optimal cross-modality integration, where CITE-seq and ASP-seq cells cluster according to biological cell types rather than sequencing modality.
(TIF)

S3 Fig The overall Macro-F1 score of CellPredX, GLUE, itClust, Portal, scJoint, scNCL, and Seurat(V5) on the matched PBMC scRNA-seq (PBMC-RNA) and scATAC-seq (PBMC-ATAC) datasets. (TIF)

S4 Fig UMAPs of PCA embeddings of the PBMC-ATAC dataset by Seurat(V5), itClust, Portal, scJoint, and scNCL, with cells colored by their cell-type annotations and prediction confidence. (TIF)

S5 Fig The decision gene expression of CD4 naïve, CD4 TCM, CD4 TEM, CD8 TEM_1, CD8 TEM_2, cDC, gdT, HSP. (TIF)

S6 Fig The decision gene expression of Intermediate B, MAIT, Memory B, Naïve B, NK, pDC, Plasma, Treg. (TIF)

S7 Fig Evaluation of Key Gene Hit Rate (KGHR) across different cell types. **(A)** The KGHR values of determinant genes for each predicted cell type in the PBMC-atac dataset. **(B)** The KGHR values of determinant genes for each predicted cell type in the ASP-seq dataset. Higher KGHR values indicate a greater overlap between determinant features identified by CellPredX and the reference marker genes, reflecting stronger biological relevance and interpretability.
(TIF)

S8 Fig Overall Macro-F1 score of CellPredX, GLUE, itClust, Portal, scJoint, scNCL and Seurat(V5) on HFA_50k, HFA_100k, and HFA_200k dataset. (TIF)

S9 Fig Heatmaps comparing the original labels and the transferred labels by Portal, scJoint, and Seurat(V5). (TIF)

S10 Fig A violin plot for the determinant genes for the part of HFA_200k dataset. (TIF)

S11 Fig Benchmarking different methods for cell type transfer. **(A)** Overall Macro-F1 score for scArches, scATAnno, scJoint, itClust, scNCL, and CellPredX across three mouse brain datasets. Each box summarizes six data points corresponding to all pairwise label transfer directions (i.e., each dataset used once as reference and once as query); **(B)** Overall Macro-F1 score of CellPredX when using different input representations of scATAC-seq data. It highlights how the choice of input format affects model performance.
(TIF)

S12 Fig The expression profile of determinant peaks on the mouse brain dataset by (A) Cusanovich et al. and (B) Fang et al. (TIF)

S13 Fig Overall Macro-F1 score of CellPredX, itClust, scArches, scGPT, scJoint, scANVI, SingleCellNet, SingleR, Seurat(V5) and TOSICA on nine human pancreas datasets. (TIF)

S14 Fig Heatmaps comparing the original labels and the transferred labels from itClust, scArches, scGPT, scJoint, Seurat(V5), and TOSICA. (TIF)

S15 Fig River plots illustrating the predicted cell types by TOSICA, Seurat(V5), scJoint, scGPT, and itClust, and their correlations to the actual cell types in the query dataset. (TIF)

S16 Fig UMAP of embeddings of the query dataset from Seurat(V5) and TOSICA. (TIF)

S17 Fig Efficiency and resource comparison of three methods on the HFA single-cell datasets. **(A)** Training time (s); **(B)** Prediction time (s); **(C)** Peak GPU memory (GB).
(TIF)

S18 Fig Parameter sensitivity experiments on HFA_50K, PBMC, mouse brain, and pancreas datasets. (TIF)

S1 Table Details of the benchmark dataset. (XLSX)

S1 Text Ablation study. (DOCX)

S2 Text Runtime and scalability analysis. (DOCX)

S3 Text Sensitivity analysis of CellPredX under different experimental settings. (DOCX)

S4 Text Comparison method parameter settings. (DOCX)

S5 Text Evaluating the performance of cell type annotation. (DOCX)
