# Supplementary figures and images for: CellPredX, a computational framework for cross-data type, cross-sample, and cross-protocol cell type annotation through domain adaptation and deep metric learning
Source: PLoS Comput Biol. 2026 Jan 2;22(1):e1013824. doi: 10.1371/journal.pcbi.1013824 (PMC12758788; doi:10.1371/journal.pcbi.1013824)

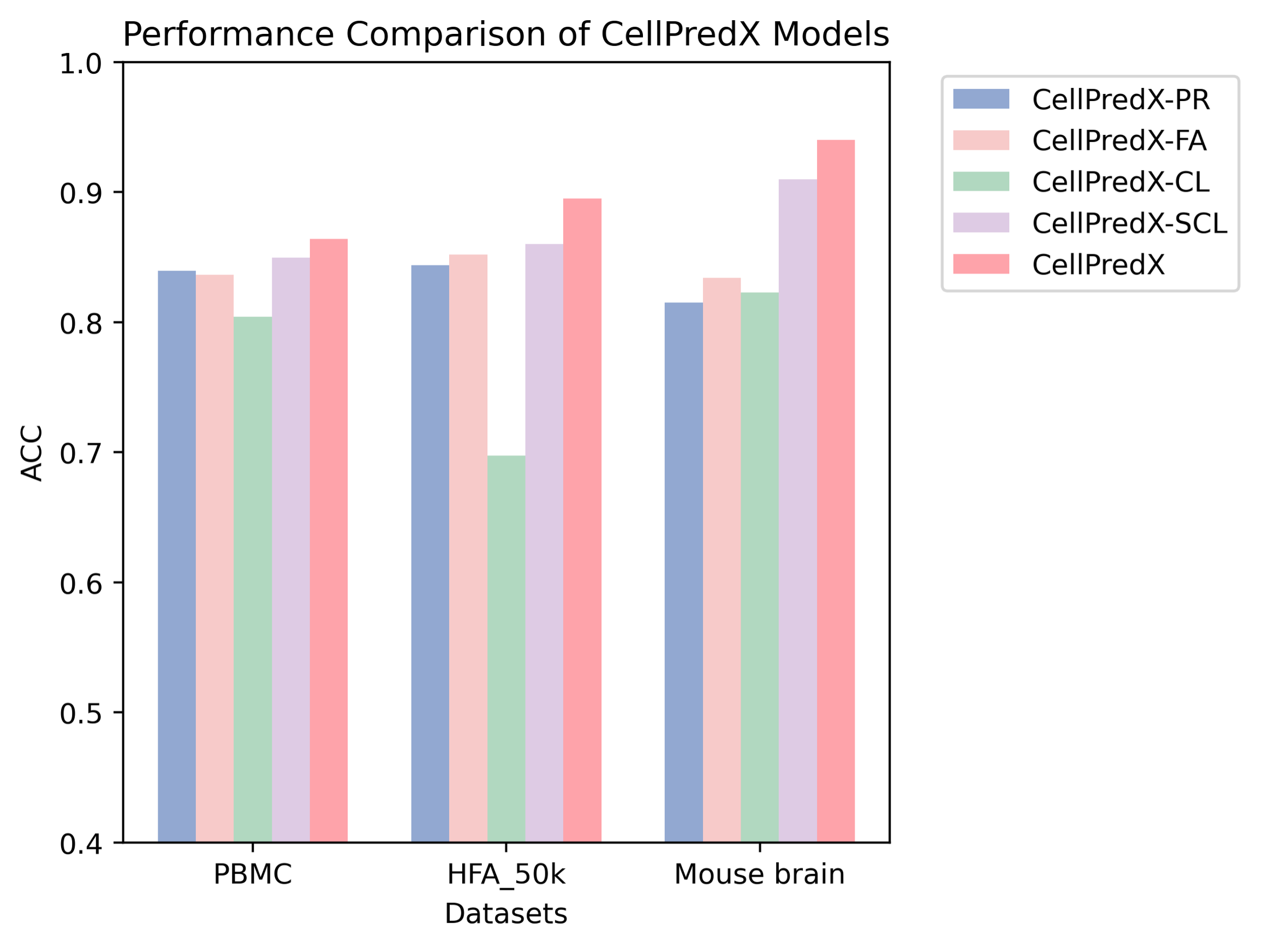

Supplement: S1 Fig — (TIF) [file pcbi.1013824.s001.tif]

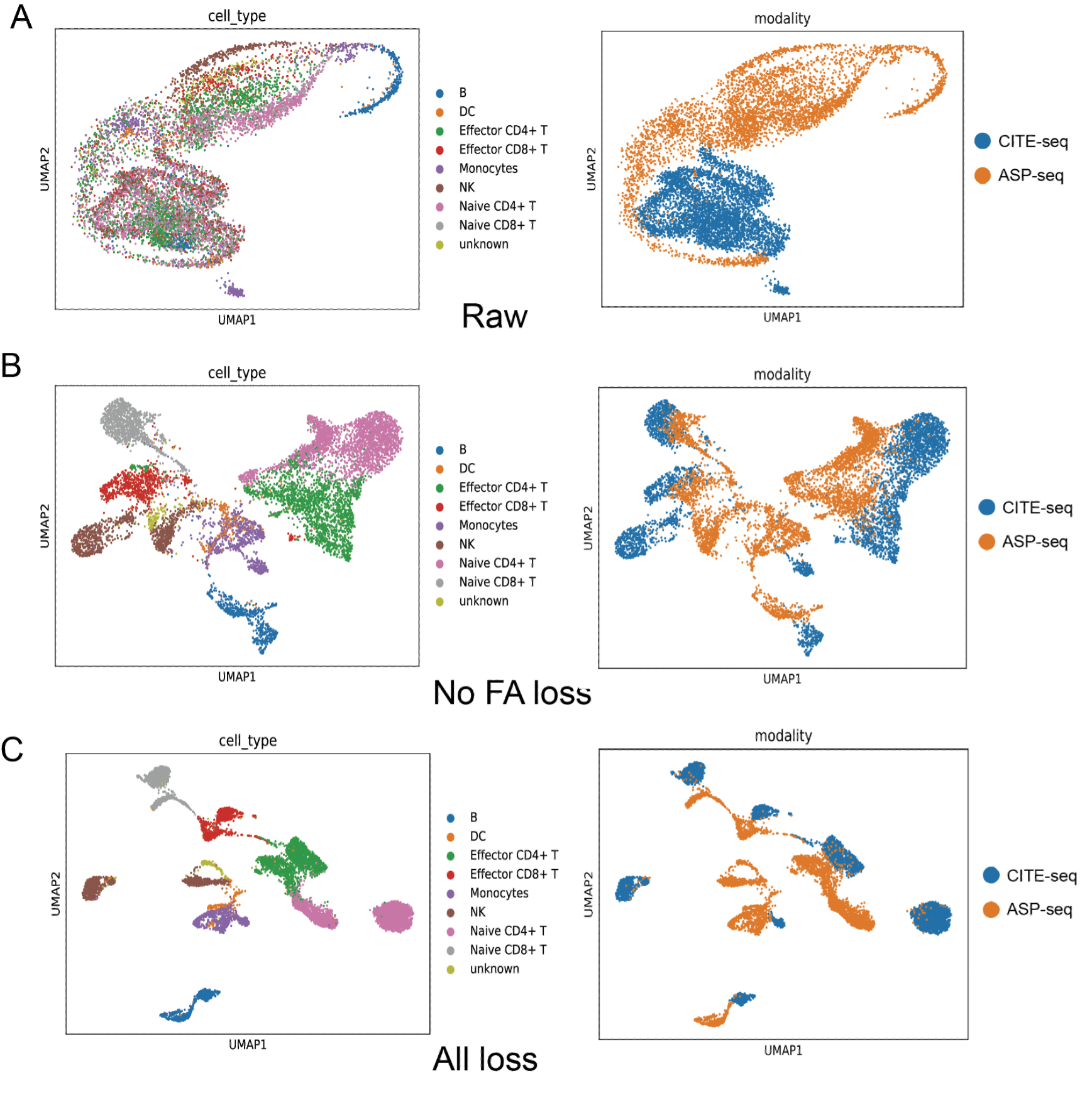

Supplement: S2 Fig — (A) Raw embeddings show strong modality-driven separation. (B) Embeddings trained without FA loss (“No FA loss”) show partial alignment but still retain modality bias. (C) Embeddings trained with all loss components (“All loss”) achieve optimal cross-modality integration, where CITE-seq and ASP-seq cells cluster according to biological cell types rather than sequencing modality. (TIF) [file pcbi.1013824.s002.tif]

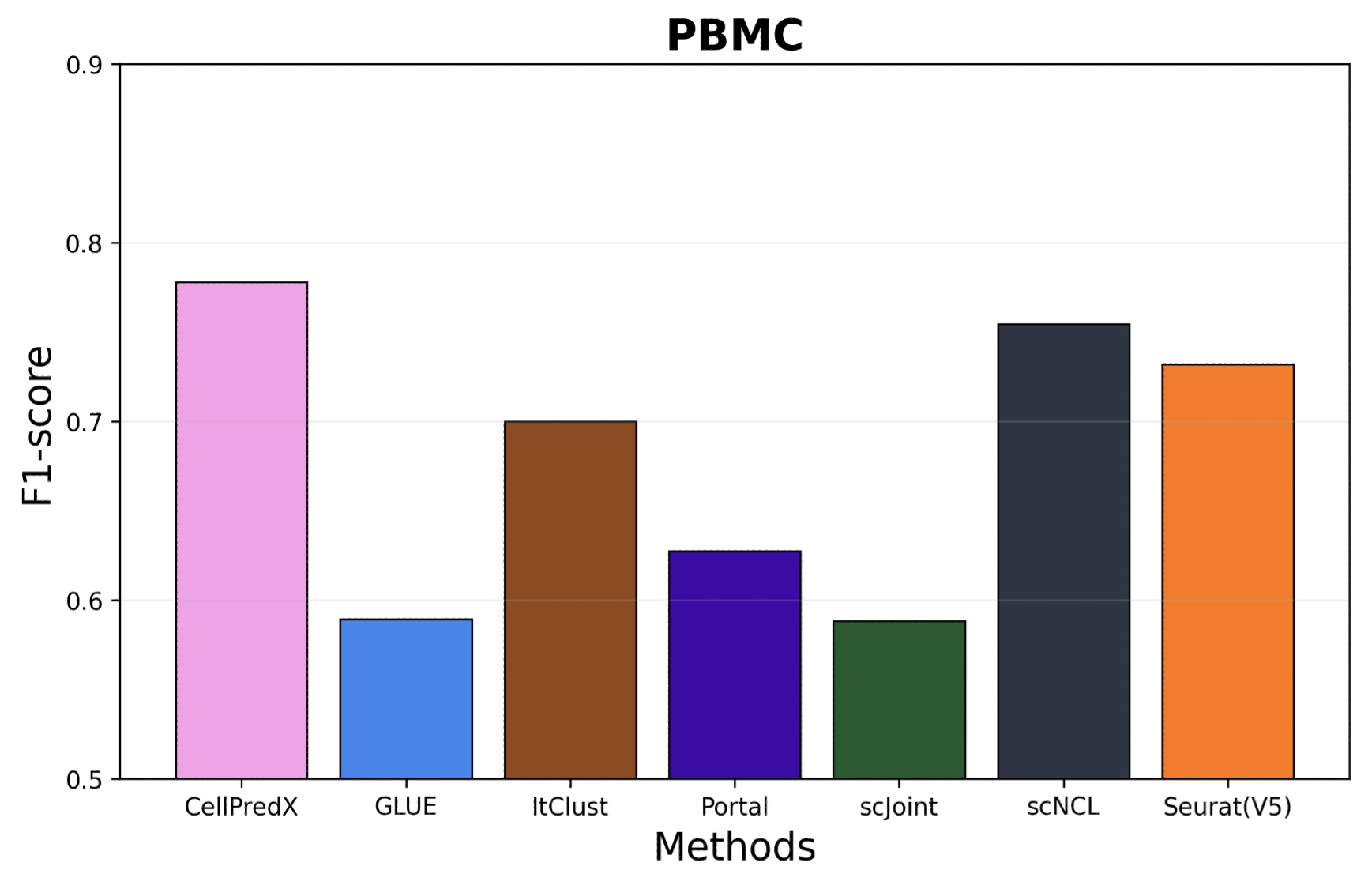

Supplement: S3 Fig — (TIF) [file pcbi.1013824.s003.tif]

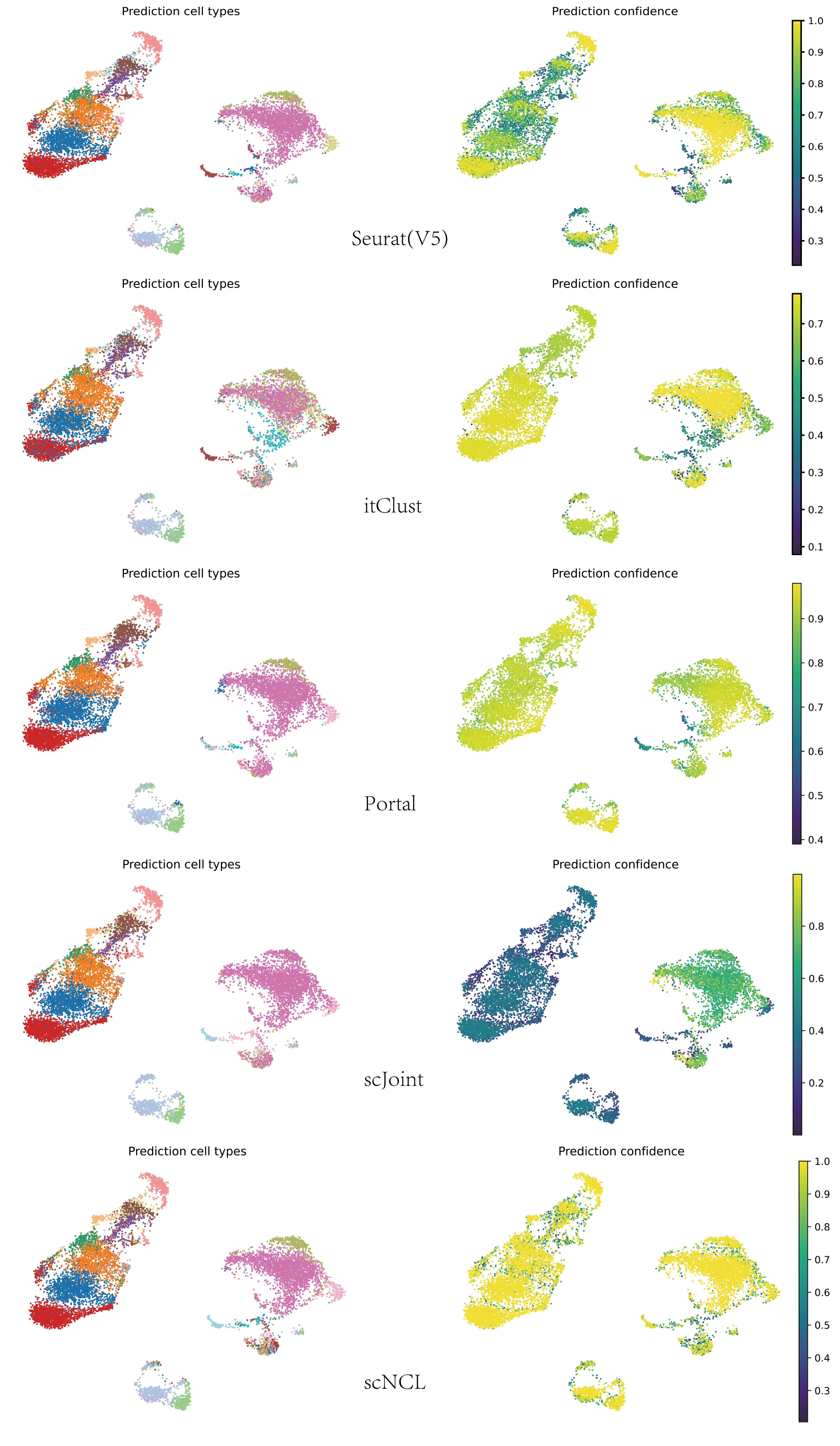

Supplement: S4 Fig — (TIF) [file pcbi.1013824.s004.tif]

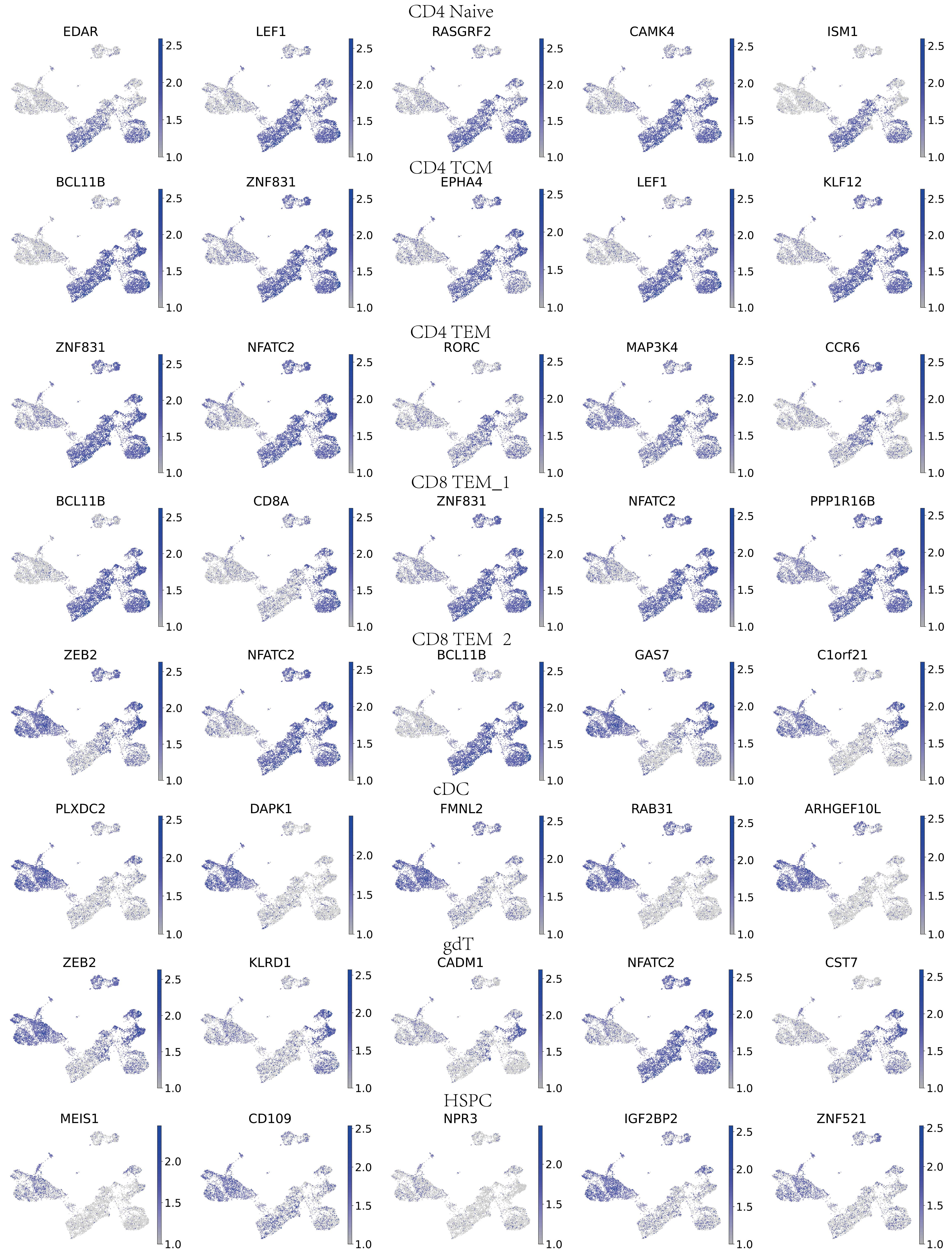

Supplement: S5 Fig — (TIF) [file pcbi.1013824.s005.tif]

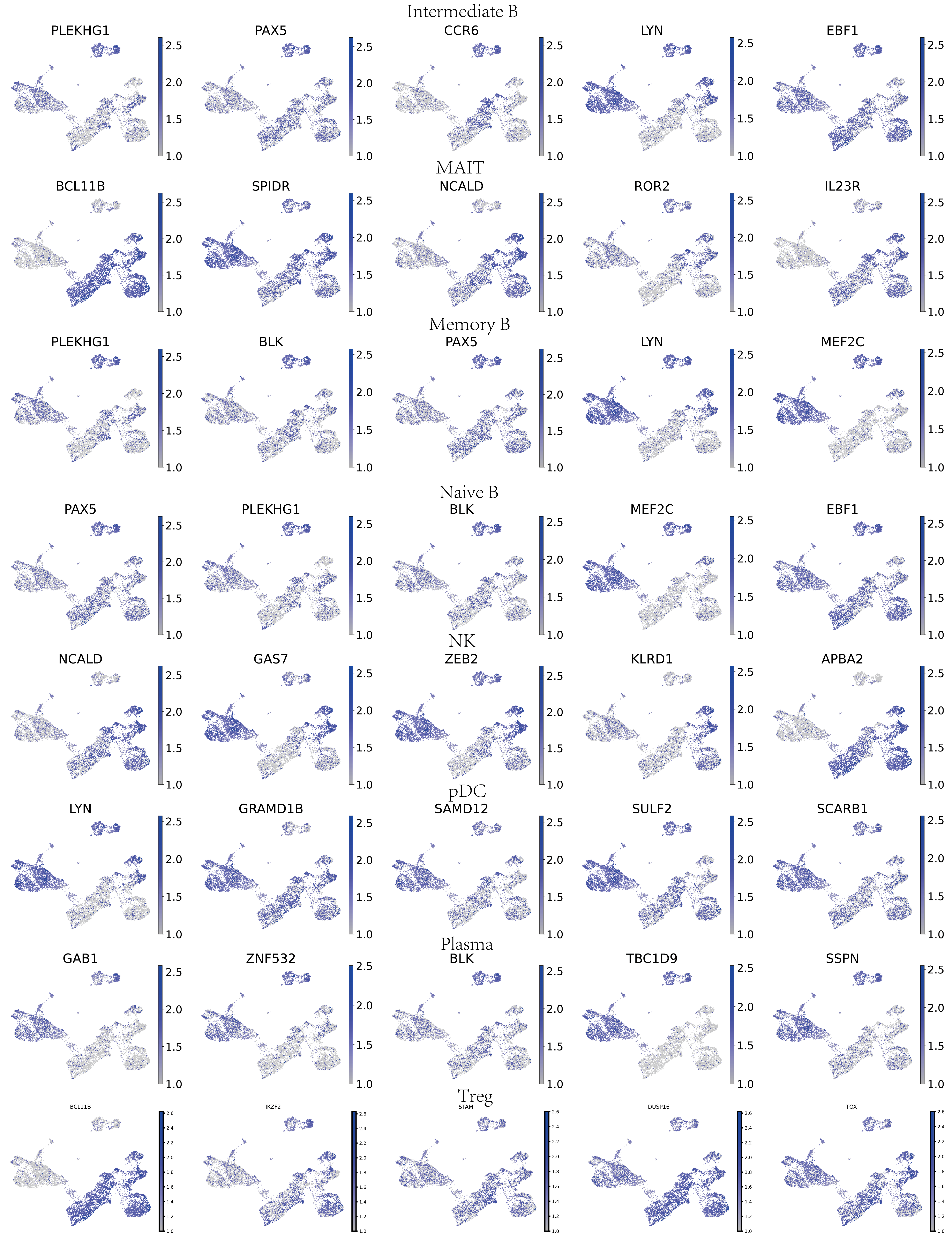

Supplement: S6 Fig — (TIF) [file pcbi.1013824.s006.tif]

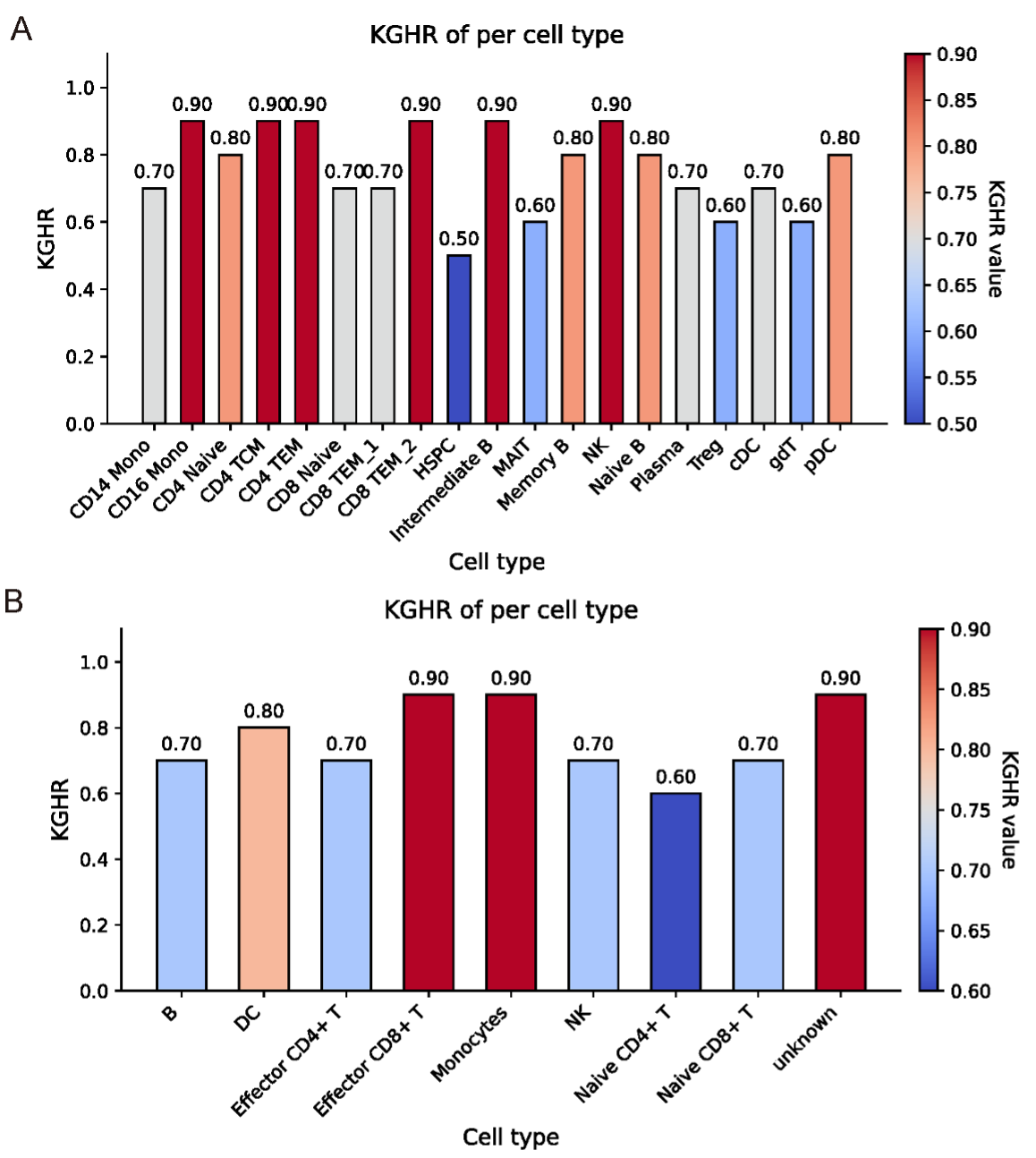

Supplement: S7 Fig — (A) The KGHR values of determinant genes for each predicted cell type in the PBMC-atac dataset. (B) The KGHR values of determinant genes for each predicted cell type in the ASP-seq dataset. Higher KGHR values indicate a greater overlap between determinant features identified by CellPredX and the reference marker genes, reflecting stronger biological relevance and interpretability. (TIF) [file pcbi.1013824.s007.tif]

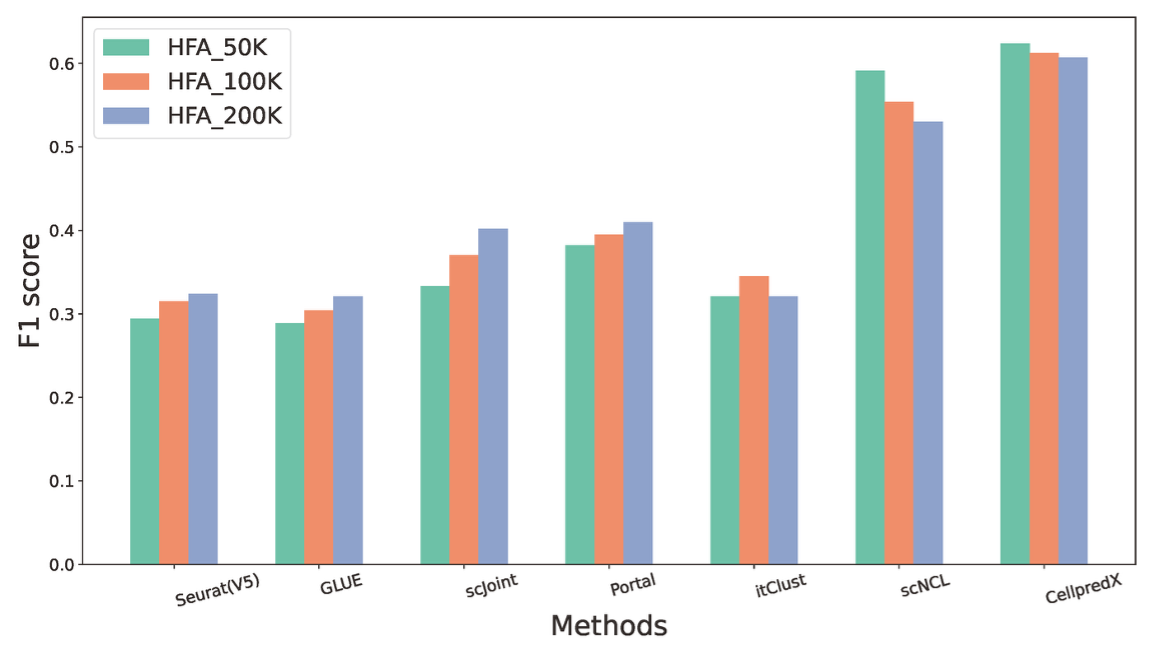

Supplement: S8 Fig — (TIF) [file pcbi.1013824.s008.tif]

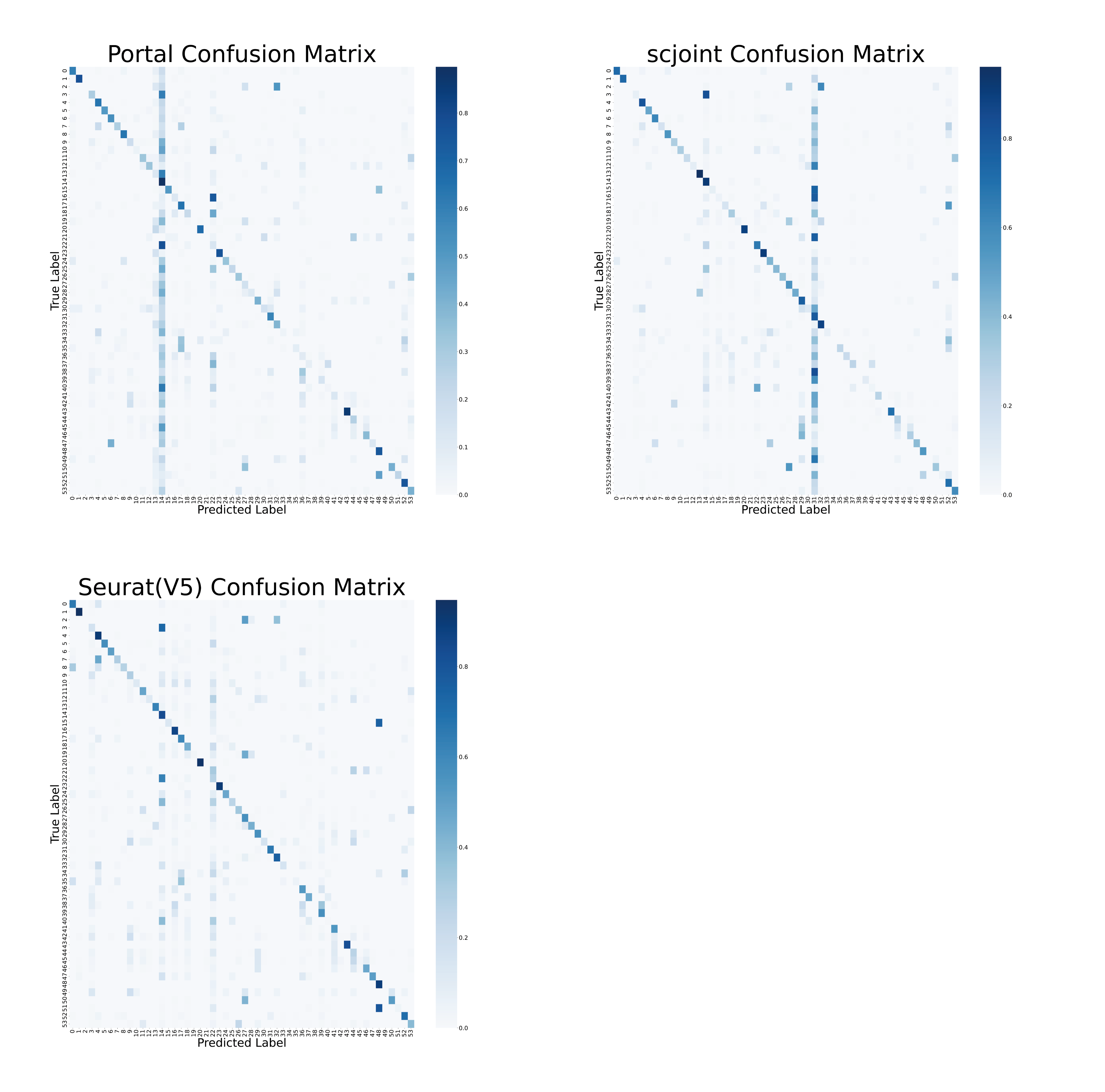

Supplement: S9 Fig — (TIF) [file pcbi.1013824.s009.tif]

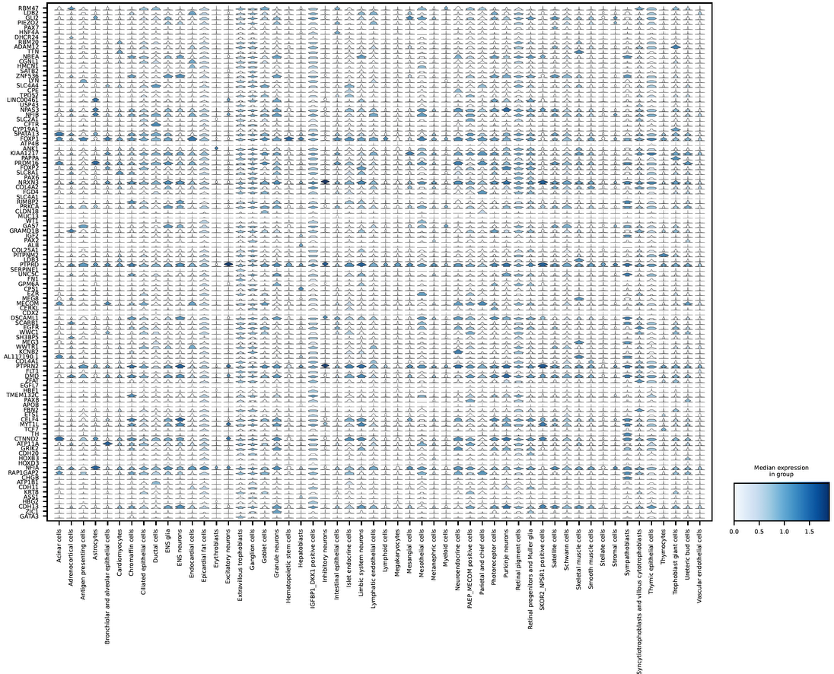

Supplement: S10 Fig — (TIF) [file pcbi.1013824.s010.tif]

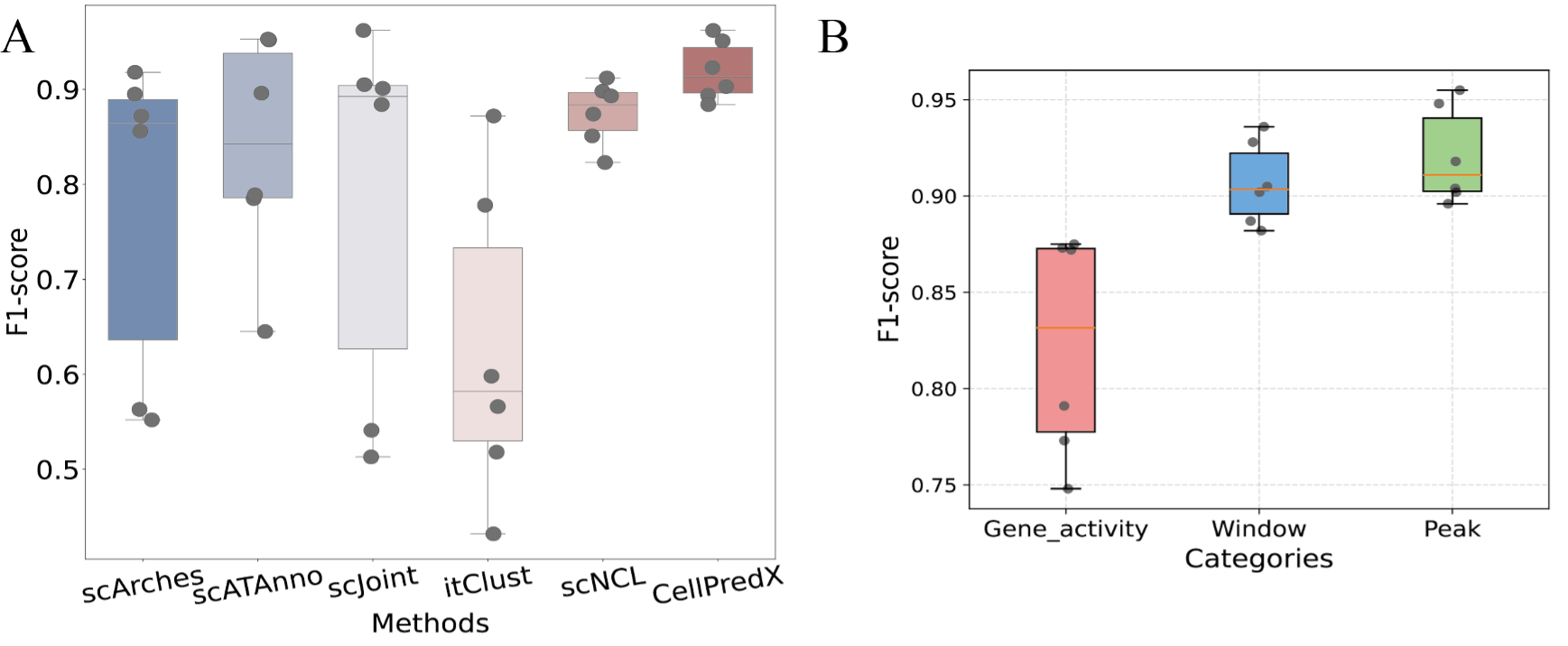

Supplement: S11 Fig — (A) Overall Macro-F1 score for scArches, scATAnno, scJoint, itClust, scNCL, and CellPredX across three mouse brain datasets. Each box summarizes six data points corresponding to all pairwise label transfer directions (i.e., each dataset used once as reference and once as query); (B) Overall Macro-F1 score of CellPredX when using different input representations of scATAC-seq data. It highlights how the choice of input format affects model performance. (TIF) [file pcbi.1013824.s011.tif]

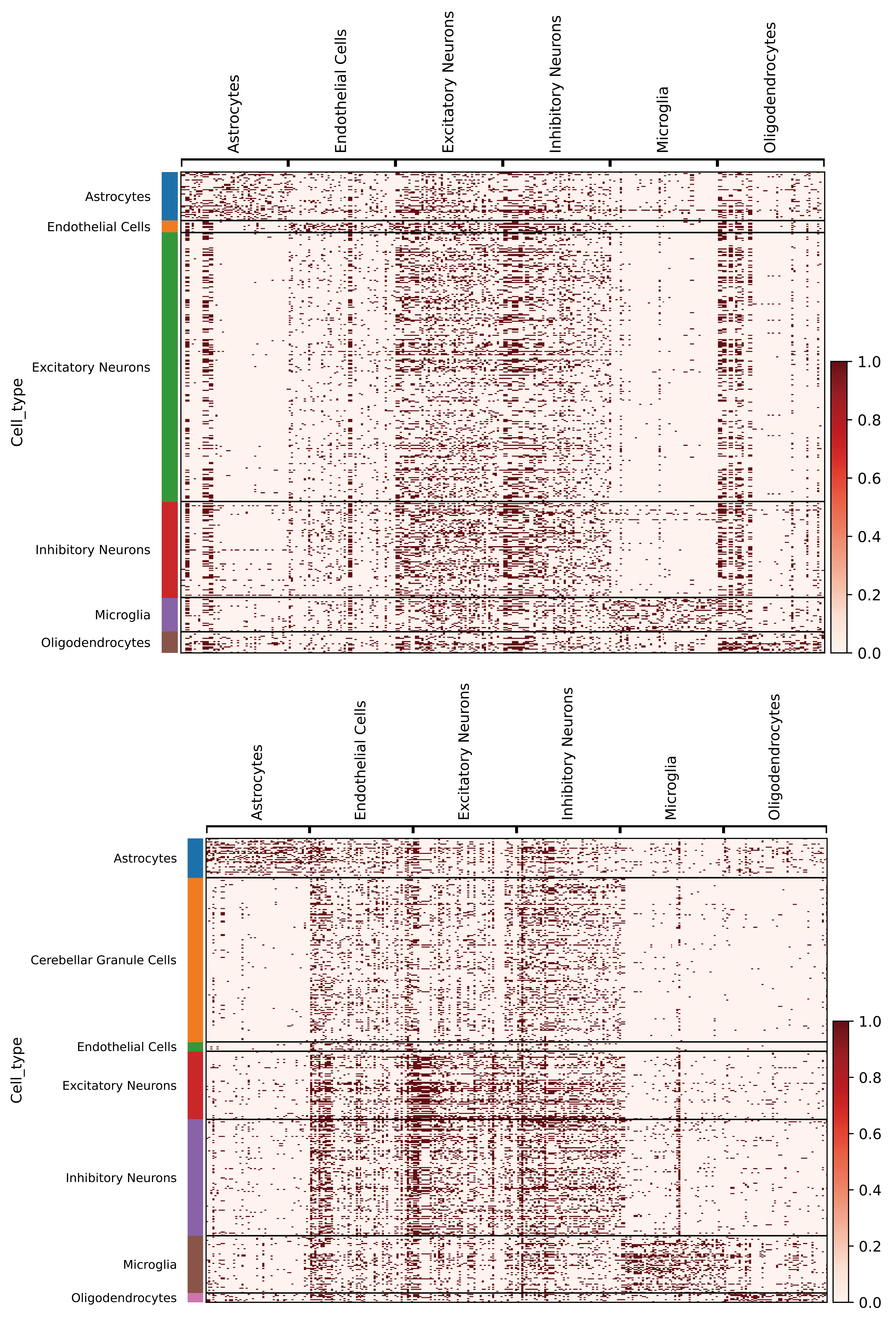

Supplement: S12 Fig — (TIF) [file pcbi.1013824.s012.tif]

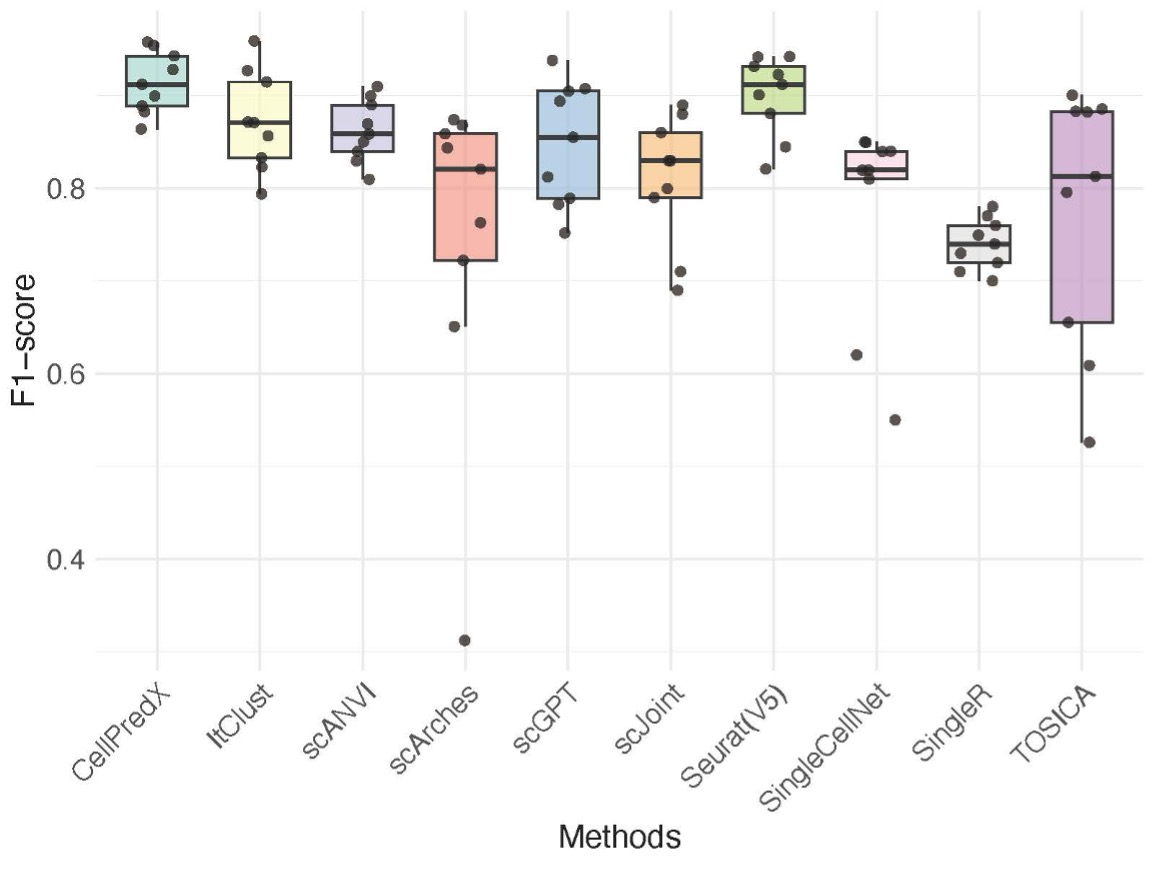

Supplement: S13 Fig — (TIF) [file pcbi.1013824.s013.tif]

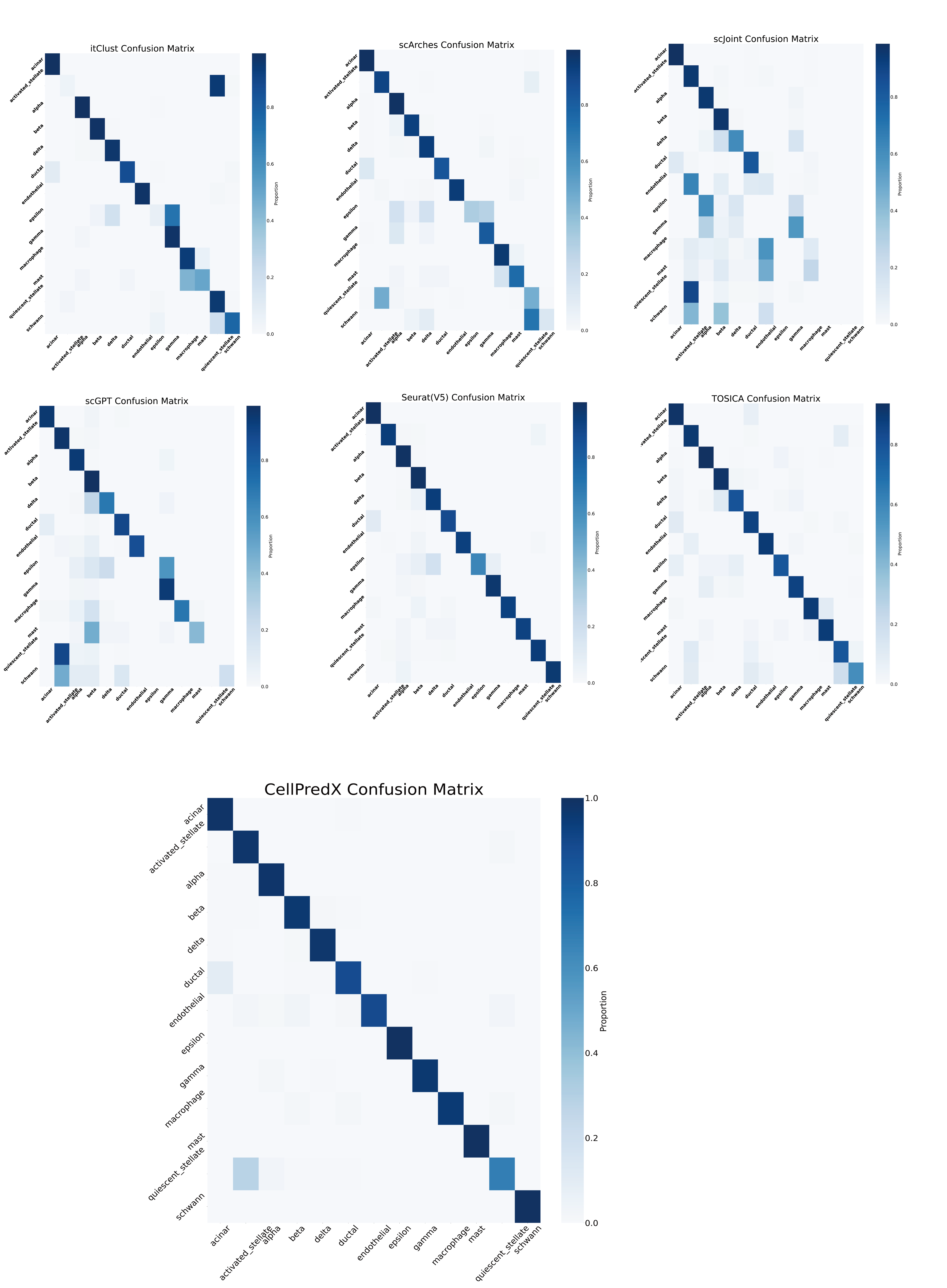

Supplement: S14 Fig — (TIF) [file pcbi.1013824.s014.tif]

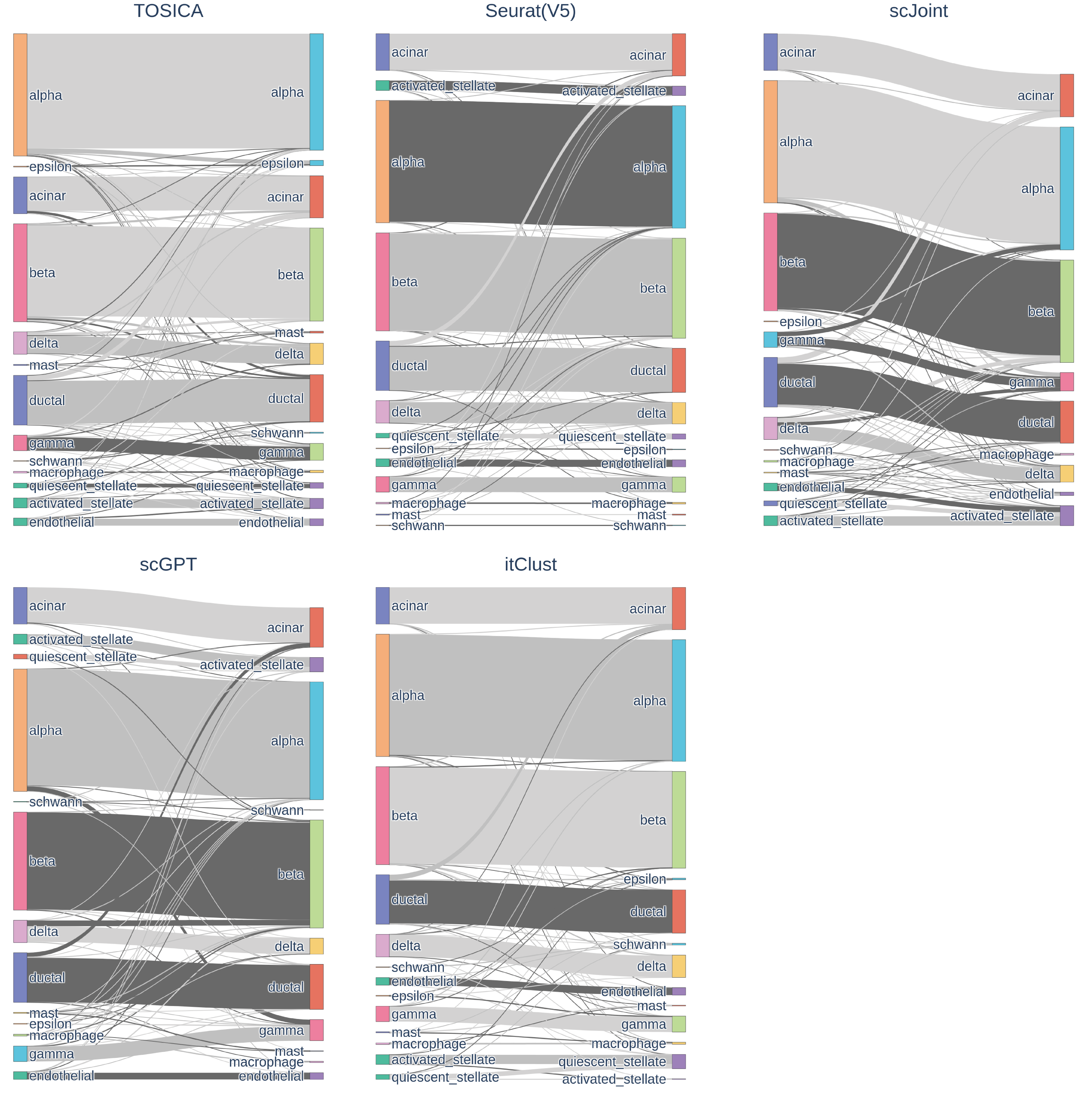

Supplement: S15 Fig — (TIF) [file pcbi.1013824.s015.tif]

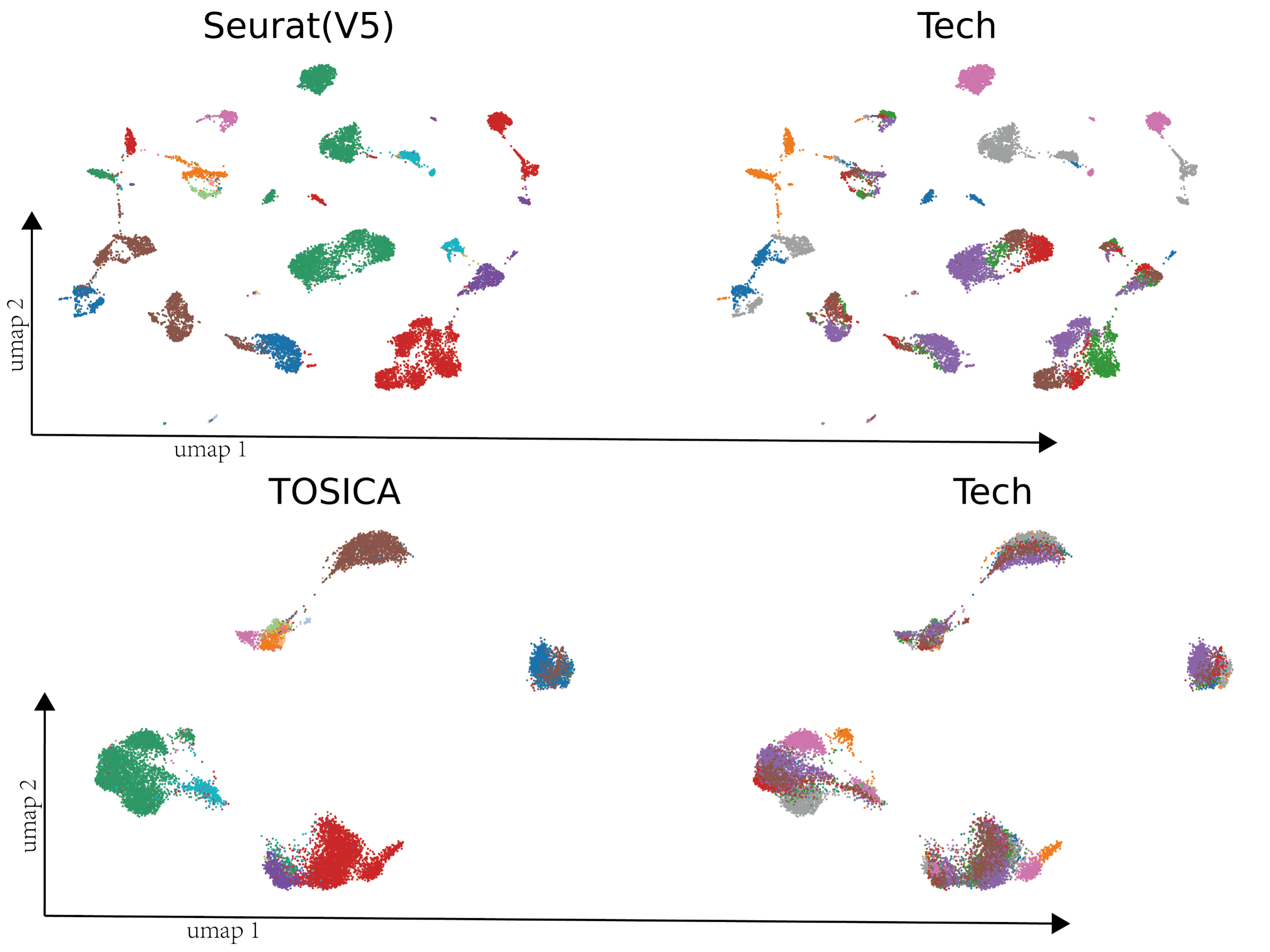

Supplement: S16 Fig — (TIF) [file pcbi.1013824.s016.tif]

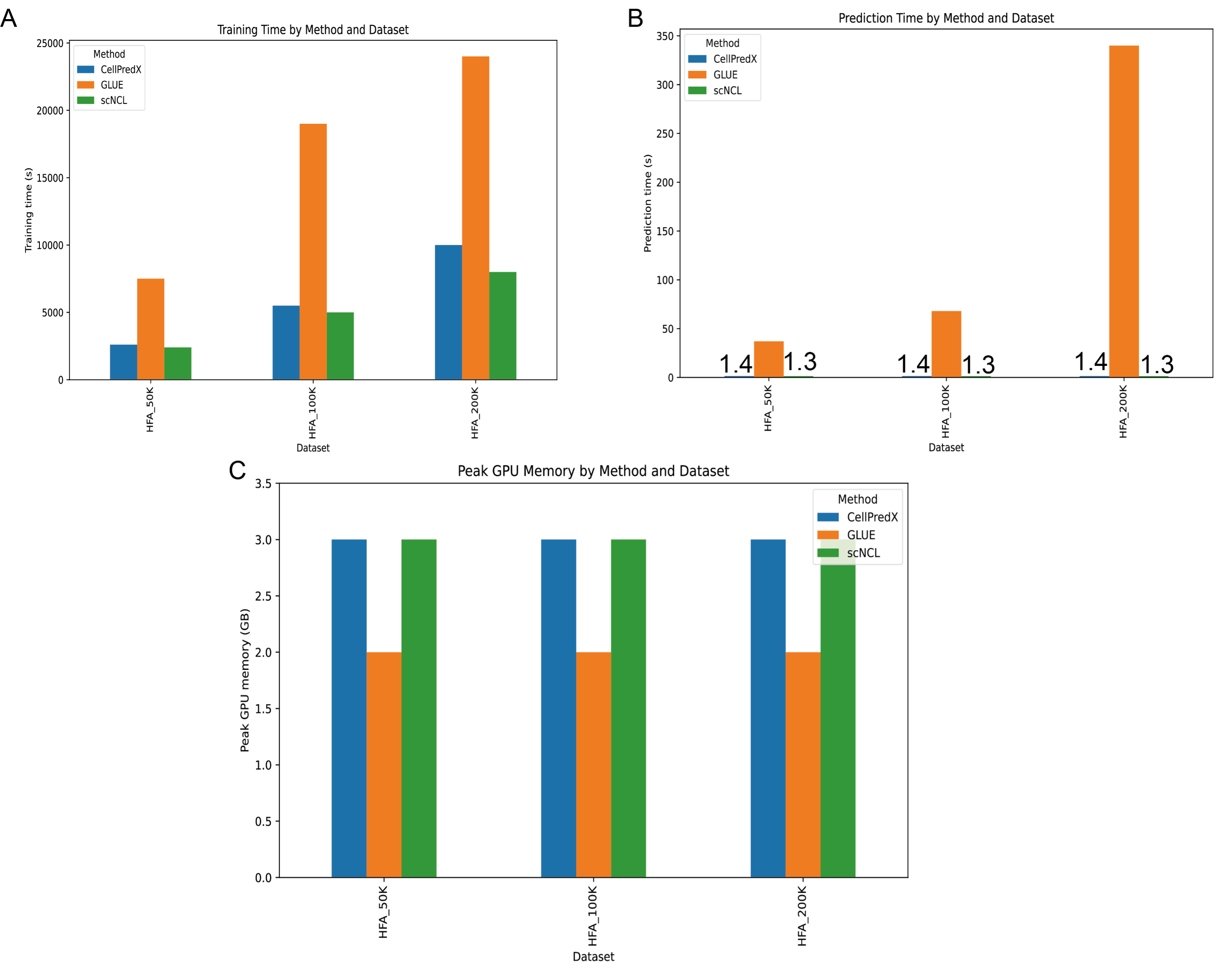

Supplement: S17 Fig — (A) Training time (s); (B) Prediction time (s); (C) Peak GPU memory (GB). (TIF) [file pcbi.1013824.s017.tif]

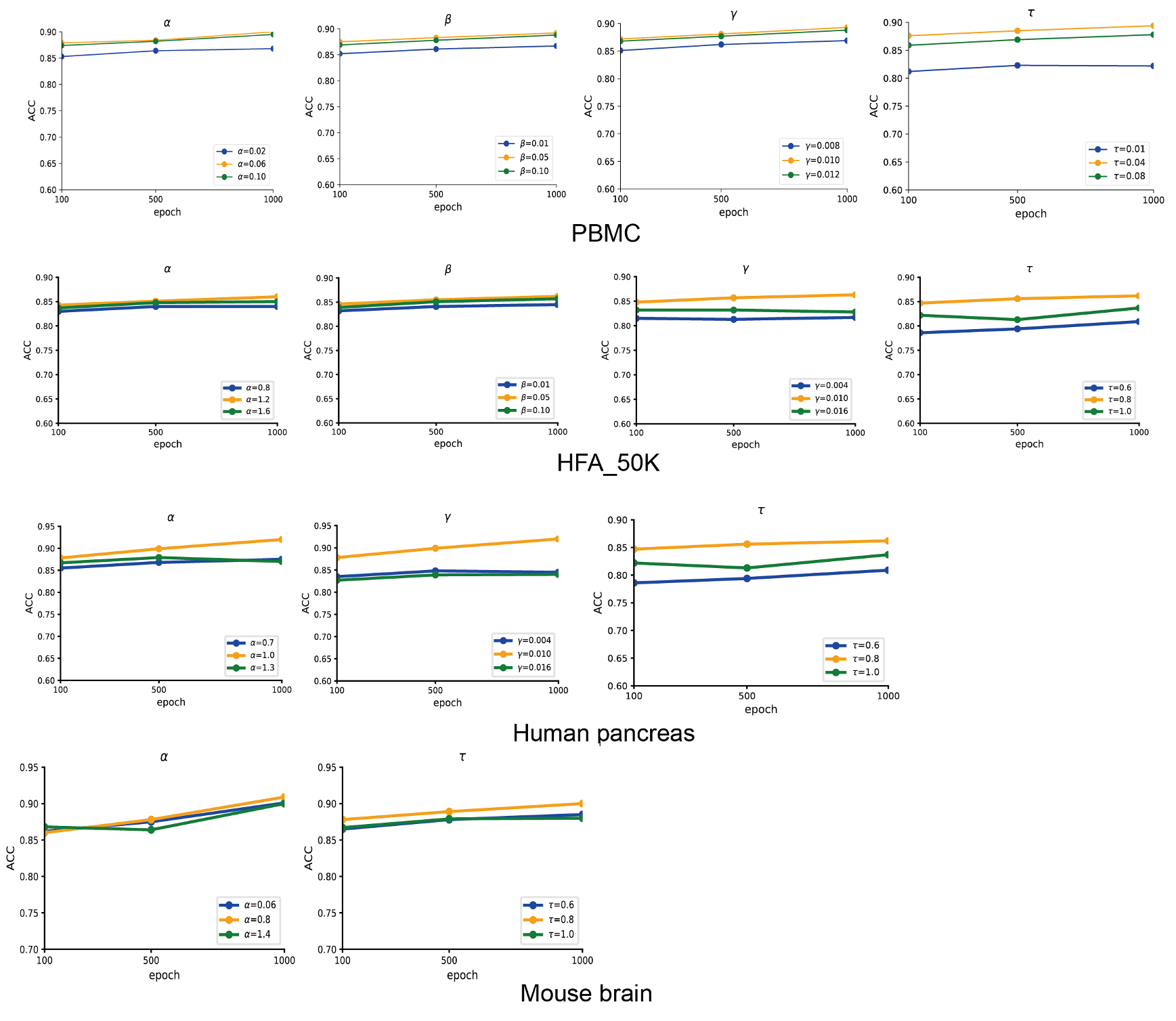

Supplement: S18 Fig — (TIF) [file pcbi.1013824.s018.tif]
