# Supplementary material for: CellPredX, a computational framework for cross-data type, cross-sample, and cross-protocol cell type annotation through domain adaptation and deep metric learning
Source: PLoS Comput Biol. 2026 Jan 2;22(1):e1013824. doi: 10.1371/journal.pcbi.1013824 (PMC12758788; doi:10.1371/journal.pcbi.1013824)
Supplement: S1 Text — (DOCX) [file pcbi.1013824.s020.docx]

**S1 Text. Ablation study**

We sought to elucidate the contribution of the individual loss functions to the enhanced performance of CellPredX. To achieve this, we conducted an ablation study on two datasets, PBMC and HFA_50k. The study comprised evaluating two distinct approaches: (1) CellPredX-PR, which employed the same loss functions as CellPredX except for the PR loss; (2) CellPredX-FA, which employed the same loss functions as CellPredX except for the FA loss; (3) CellPredX-CL, which employed the same loss functions as CellPredX except for the CL loss; and (4) CellPredX-SCL, which employed the same loss functions as CellPredX except for the SCL loss. The outcomes of the ablation study are documented in **S1 Fig**. Analysis of the results reveals a marked decline in transfer accuracy across the two datasets when individual loss functions were omitted. Therefore, the loss functions used all contributed to CellPredX’s superior performance. This insight suggests that these components are integral to the model's ability to handle complex variations in data, ultimately facilitating robust and accurate cell type annotation in diverse experimental settings.

To further illustrate the effect of the FA loss on cross-modality integration, we visualized the embeddings of CITE-seq and ASP-seq data before and after applying the FA loss. As shown in **S2 Fig**, the raw embeddings exhibited clear modality separation, indicating limited alignment between CITE-seq and ASP-seq cells. When trained without the FA loss, the integration improved moderately, yet modality-specific clusters remained distinct. In contrast, incorporating the FA loss led to well-aligned and biologically coherent clusters, where cells from both modalities were mixed according to their cell types rather than sequencing technologies. These results demonstrate that the FA loss effectively reduces modality gaps and promotes consistent feature alignment across datasets.
