# Supplementary material for: CellPredX, a computational framework for cross-data type, cross-sample, and cross-protocol cell type annotation through domain adaptation and deep metric learning
Source: PLoS Comput Biol. 2026 Jan 2;22(1):e1013824. doi: 10.1371/journal.pcbi.1013824 (PMC12758788; doi:10.1371/journal.pcbi.1013824)
Supplement: S2 Text — (DOCX) [file pcbi.1013824.s021.docx]

**S2 Text. Runtime and Scalability Analysis**

To evaluate the runtime and scalability of CellPredX, we compared it with GLUE and scNCL on three dataset sizes: HFA_50k, HFA_100k, and HFA_200k. Across all sizes (**S17 Fig**), GLUE recorded the longest wall clock time, primarily because its pipeline constructs a global kNN neighbor graph and performs cross dataset alignment. As the reference set increased, the cost of graph construction and querying scaled at least linearly with the number of cells. Consequently, the training time increased from about 7,500 seconds to about 19,000 and 24,000 seconds. For a fixed query set of 10,000 cells, the prediction time rose from about 37 seconds to about 68 and 340 seconds. Peak GPU memory remained modest at about 2.0 GB, since the dominant costs involved neighbor search and graph bookkeeping rather than deep model activations.

In contrast, CellPredX avoids a global neighbor graph during both training and inference. Computation is dominated by mini batch forward and backward passes with a fixed size model, which yields nearly linear scaling with sample size. The training time was about 2,600 seconds for 50,000 cells, about 5,500 seconds for 100,000 cells, and about 10,000 seconds for 200,000 cells. For a held out query set of 10,000 cells, the prediction time was essentially constant at about 1.4 seconds because inference requires a single forward pass without recomputing any reference graph. Peak GPU memory was stable at about 3.0 GB across scales, reflecting batch bounded activations and an inference path that does not depend on reference size.

Compared with scNCL under the same settings, both methods avoid a global neighbor graph and scale linearly or nearly linearly in training and inference; however, scNCL was faster overall. The training time was about 2,400 seconds for 50,000 cells, about 5,000 seconds for 100,000 cells, and about 8,000 seconds for 200,000 cells, which corresponds to an improvement of approximately seven to twenty percent relative to CellPredX. Prediction on the 10, 000 cell query set was about 1.3 seconds for scNCL and about 1.4 seconds for CellPredX. Peak GPU memory for both methods was about 3.0 GB and essentially invariant to dataset size.

Overall, for datasets with at least 100,000 cells, both CellPredX and scNCL provide deployable linear scaling and near instant inference latency. Considering accuracy together with efficiency, CellPredX is a fast and accurate method for scATAC-seq cell type annotation.
