# Supplementary material for: CellPredX, a computational framework for cross-data type, cross-sample, and cross-protocol cell type annotation through domain adaptation and deep metric learning
Source: PLoS Comput Biol. 2026 Jan 2;22(1):e1013824. doi: 10.1371/journal.pcbi.1013824 (PMC12758788; doi:10.1371/journal.pcbi.1013824)
Supplement: S3 Text — (DOCX) [file pcbi.1013824.s022.docx]

**S3 Text. Sensitivity Analysis of CellPredX under Different Experimental Settings**

We evaluated the sensitivity of CellPredX to different parameter settings across multiple experimental scenarios, including unmatched scATAC-seq cell type annotation, matched scATAC-seq cell type annotation, label transfer between scATAC-seq datasets, and cell type transfer using scRNA-seq datasets. The datasets used for these experiments include HFA_50K, PBMC, mouse brain, and pancreas datasets. Specifically, for the mouse brain experiments, mouse brain-Fang was used as the reference dataset and mouse brain-10X as the query dataset; for the pancreas experiments, Human pancreas (inDrop1) was used as the reference dataset and Human pancreas (inDrop2) as the query dataset. All experiments were conducted using the default parameter settings, and the evaluation results are presented in **S18 Fig**.

Overall, CellPredX demonstrates strong robustness to parameter variations across most scenarios. In unmatched and matched scATAC-seq cell type annotation tasks, CellPredX is insensitive to both α and β. In cell type transfer using scRNA-seq datasets, CellPredX is insensitive to α and γ. In contrast, during label transfer between scATAC-seq datasets, CellPredX shows relatively higher sensitivity to α.

Across all scenarios, τ has a relatively important impact on CellPredX’s performance, and the optimal value of τ varies across datasets. Nevertheless, we found that τ = 0.8 consistently yields stable and high performance, except for the matched scRNA-seq and scATAC-seq setting. For α, a relatively small value (around 0.06) works well in the matched scRNA-seq and scATAC-seq integration scenario, whereas a larger value (around 1.2) is recommended for the unmatched case. For the other two scenarios, α = 0.1 produces stable results. Parameters β and γ mainly serve as regularization terms, and their values are recommended to be kept below 0.1.

Since CellPredX provides a unified framework for solving four distinct types of cell type annotation problems, parameter tuning may be required to account for modality discrepancies and batch effects of different datasets. However, our experiments indicate that the parameter ranges are well-controlled and easy to adjust, demonstrating that CellPredX is a robust and versatile tool for cell type annotation across diverse single-cell multi-omics scenarios.
