# Supplementary material for: CellPredX, a computational framework for cross-data type, cross-sample, and cross-protocol cell type annotation through domain adaptation and deep metric learning
Source: PLoS Comput Biol. 2026 Jan 2;22(1):e1013824. doi: 10.1371/journal.pcbi.1013824 (PMC12758788; doi:10.1371/journal.pcbi.1013824)
Supplement: S4 Text — (DOCX) [file pcbi.1013824.s023.docx]

**S4 Text. Comparison Method Parameter Settings**

**Seurat(V5) [1]:** The R package Seurat v.5.0.1 was utilized for the selected datasets. For label propagation from scRNA-seq to scATAC-seq data, the gene expression matrix (GEM) of scRNA-seq and the gene activity matrix (GAM) of scATAC-seq were used as inputs and normalized using the ‘NormalizeData’ function. The ‘FindVariableFeatures’ function, employing the ‘vst’ method, was used to select the top 2000 most variable genes from the scRNA-seq data. The ‘FindTransferAnchors’ function, with 'cca' reduction, was then used to identify anchors between the scRNA-seq and scATAC-seq data. Finally, the ‘TransferData’ function was utilized to transfer annotations from scRNA-seq to scATAC-seq. For the label transfer between scRNA-seq data, the GEM were used as the inputs and normalized using the ‘NormalizeData’ function. The settings remained consistent with the aforementioned procedure, except for the ‘FindTransferAnchors’ function, which utilized 'pca' reduction, retaining 30 principal components. The prediction confidence $p$can be directly derived from the transfer results. The confidence that a cell was classified as a novel type was determined by calculating $1-p$.

**Portal [2]:** The Python package Portal v1.0.2 was employed for the selected datasets. The GEM of scRNA-seq and the GAM of scATAC-seq were used as inputs and normalized using the ‘NormalizeData’ function. The model was configured with the parameters including training_steps=2000, lambdacos=10, lambdaAE=10, lambdaLA=10, and lambdaGAN=1.0. A *K*-Nearest Neighbors (KNN) classifier, with $K=10$, was utilized to annotate the query dataset. The output provided the prediction confidence $p$, and the confidence that a cell was identified as a novel cell type was determined by calculating $1-p$.

**GLUE [3]:** The Python package GLUE v 0.3.2 was utilized for the selected datasets. The GEM of scRNA-seq and the GAM of scATAC-seq were used as inputs and normalized using the ‘NormalizeData’ function. GEM and GAM were downscaled to 100 dimensions using the Principal Component Analysis (PCA). The ‘scglue.data.get_gene_annotation’ function was employed to extract the coordinate information of genes from the GTF file. The ‘scglue.genomics.rna_anchored_guidance_graph’ function was used to construct the graph adjacency matrix, utilizing the default parameters of ‘scglue.model.configure_dataset’ to set up the training and test datasets. Upon completing model training, we used the ‘scglue.data.transfer_labels’ function to perform cell type annotation.

**itClust [4]:** The Python package itClust v1.2.0 was used for the selected datasets. For label propagation from scRNA-seq to scATAC-seq, the GEM and GAM were used as inputs. For label propagation between scATAC-seq datasets, peak matrices of scATAC-seq data were used as inputs. For label propagation between scRNA-seq datasets, the GEMs were used as inputs. The default parameters of the ‘transfer_learning_clf’ function were used to train the model, and the ‘clf.predict’ function was employed to annotate the query dataset.

**scJoint [5]:** The Python package scJoint was utilized for the selected datasets, For label propagation from scRNA-seq to scATAC-seq, the GEM and GAM were used as inputs. For label propagation between scATAC-seq datasets, peak matrices of scATAC-seq data were used as inputs. For label propagation between scRNA-seq datasets, the GEMs were used as inputs. The training parameters are set to the default used in their GitHub repository (URL:<https://github.com/SydneyBioX/scJoint>), The output provided the prediction confidence $p$, and the confidence for a cell being identified as a novel cell type was determined by calculating $1-p$.

**scNCL [6]:** The Python package scNCL was utilized for the selected datasets, for label propagation from scRNA-seq to scATAC-seq, the GEM and GAM were used as inputs. For label propagation between scATAC-seq datasets, peak matrices of scATAC-seq data were used as inputs. For label propagation between scRNA-seq datasets, the GEMs were used as inputs. The training parameters are set to the default used in their GitHub repository (URL:<https://github.com/CSUBioGroup/scNCL-release>), The output provided the prediction confidence $p$, and the confidence that a cell was identified as a novel cell type was determined by calculating $1-p$.

**scArches [7]:** The Python package scArches was utilized for the selected datasets, For label propagation between scATAC-seq datasets, peak matrices of scATAC-seq data were used as inputs. For label propagation between scRNA-seq datasets, the GEMs were used as inputs. The ‘scarches.models.scpoli’ import scPoli model is used to perform cell type annotation and the parameter is consistent with what their GitHub repository (URL<https://github.com/theislab/scarches>).

**scGPT [8]:** The Python package scGPT was utilized for the selected datasets. For label propagation between scRNA-seq datasets, the GEMs were used as inputs. We refer to this tutorial [(https://github.com/bowang-lab/scGPT/blob/main/tutorials/Tutorial_Annotation.ipynb)](https://github.com/bowang-lab/scGPT/blob/main/tutorials/Tutorial_Annotation.ipynb) for cell type annotations, and the parameters remain the same.

**TOSICA [9]: Similar to scGPT,** for label propagation between scRNA-seq datasets, the GEMs were used as inputs. We refer to this tutorial (<https://github.com/JackieHanLab/TOSICA/blob/main/test/tutorial.ipynb>) for parameter and cell type annotations.

**scANVI [10]**: We used scANVI (as implemented in scvi-tools [11]) for scRNA-seq cell-type annotation. Following the package’s standard preprocessing, we configured the model with n_layers = 3, n_latent = 30, and gene_likelihood = “nb”. Training was run for max_epochs = 100, using n_samples_per_label = 100 to balance mini-batches across labels; all other hyperparameters were kept at their defaults. The model’s posterior class probabilities were used to assign cell-type labels.

**SingleCellNet [12]**: We used the implementation from the Cahan Lab repository (URL:<https://github.com/CahanLab/singleCellNet>), we followed the repository’s preprocessing and marker-pair selection pipeline and set the training hyperparameters to: nTopGenes = 10, nRand = 70, nTrees = 1000, nTopGenePairs = 25, dLevel = “newAnn”, and colName_samp = “cell”.

**SingleR [13]**: For baseline comparison, we applied the SingleR method (R package version 12.18.2018) for automated cell type annotation. The implementation was obtained from the official GitHub repository (<https://github.com/dviraran/SingleR>). We followed the authors’ recommended pipeline and parameter settings as described in the original study, without additional modifications.

**CellPredX:** For label propagation from scRNA-seq to scATAC-seq, the GEM and GAM were used as inputs. For label propagation between scATAC-seq datasets, peak matrices of scATAC-seq data were used as inputs. For label propagation between scRNA-seq datasets, the GEMs were used as inputs.

**Reference**

[1] Y. Hao, T. Stuart, M. H. Kowalski, S. Choudhary, P. Hoffman, A. Hartman, A. Srivastava, G. Molla, S. Madad, and C. Fernandez-Granda, “Dictionary learning for integrative, multimodal and scalable single-cell analysis,” *Nature biotechnology,* vol. 42, no. 2, pp. 293-304, 2024.

[2] J. Zhao, G. Wang, J. Ming, Z. Lin, Y. Wang, A. R. Wu, and C. Yang, “Adversarial domain translation networks for integrating large-scale atlas-level single-cell datasets,” *Nature computational science,* vol. 2, no. 5, pp. 317-330, 2022.

[3] Z.-J. Cao, and G. Gao, “Multi-omics single-cell data integration and regulatory inference with graph-linked embedding,” *Nature Biotechnology,* vol. 40, no. 10, pp. 1458-1466, 2022.

[4] J. Hu, X. Li, G. Hu, Y. Lyu, K. Susztak, and M. Li, “Iterative transfer learning with neural network for clustering and cell type classification in single-cell RNA-seq analysis,” *Nature machine intelligence,* vol. 2, no. 10, pp. 607-618, 2020.

[5] Y. Lin, T.-Y. Wu, S. Wan, J. Y. Yang, W. H. Wong, and Y. R. Wang, “scJoint integrates atlas-scale single-cell RNA-seq and ATAC-seq data with transfer learning,” *Nature biotechnology,* vol. 40, no. 5, pp. 703-710, 2022.

[6] X. Yan, R. Zheng, J. Chen, and M. Li, “scNCL: transferring labels from scRNA-seq to scATAC-seq data with neighborhood contrastive regularization,” *Bioinformatics,* vol. 39, no. 8, pp. btad505, 2023.

[7] M. Lotfollahi, M. Naghipourfar, M. D. Luecken, M. Khajavi, M. Büttner, M. Wagenstetter, Ž. Avsec, A. Gayoso, N. Yosef, and M. Interlandi, “Mapping single-cell data to reference atlases by transfer learning,” *Nature biotechnology,* vol. 40, no. 1, pp. 121-130, 2022.

[8] H. Cui, C. Wang, H. Maan, K. Pang, F. Luo, N. Duan, and B. Wang, “scGPT: toward building a foundation model for single-cell multi-omics using generative AI,” *Nature Methods*, pp. 1-11, 2024.

[9] J. Chen, H. Xu, W. Tao, Z. Chen, Y. Zhao, and J.-D. J. Han, “Transformer for one stop interpretable cell type annotation,” *Nature Communications,* vol. 14, no. 1, pp. 223, 2023.

[10] C. Xu, R. Lopez, E. Mehlman, J. Regier, M. I. Jordan, and N. Yosef, “Probabilistic harmonization and annotation of single‐cell transcriptomics data with deep generative models,” *Molecular systems biology,* vol. 17, no. 1, pp. e9620, 2021.

[11] A. Gayoso, R. Lopez, G. Xing, P. Boyeau, V. Valiollah Pour Amiri, J. Hong, K. Wu, M. Jayasuriya, E. Mehlman, and M. Langevin, “A Python library for probabilistic analysis of single-cell omics data,” *Nature biotechnology,* vol. 40, no. 2, pp. 163-166, 2022.

[12] Y. Tan, and P. Cahan, “SingleCellNet: a computational tool to classify single cell RNA-Seq data across platforms and across species,” *Cell systems,* vol. 9, no. 2, pp. 207-213. e2, 2019.

[13] D. Aran, A. P. Looney, L. Liu, E. Wu, V. Fong, A. Hsu, S. Chak, R. P. Naikawadi, P. J. Wolters, and A. R. Abate, “Reference-based analysis of lung single-cell sequencing reveals a transitional profibrotic macrophage,” *Nature immunology,* vol. 20, no. 2, pp. 163-172, 2019.
