# Supplementary material for: CellPredX, a computational framework for cross-data type, cross-sample, and cross-protocol cell type annotation through domain adaptation and deep metric learning
Source: PLoS Comput Biol. 2026 Jan 2;22(1):e1013824. doi: 10.1371/journal.pcbi.1013824 (PMC12758788; doi:10.1371/journal.pcbi.1013824)
Supplement: S5 Text — (DOCX) [file pcbi.1013824.s024.docx]

**S5 Text. Evaluating the performance of cell type annotation**

We evaluate the cell type annotation performance of CellPredX in two ways: (1) the ability to predict shared cell types between reference and query datasets; and (2) the performance of detecting novel cell types. We used accuracy and macro-F1 to assess the ability of CellPredX to predict shared cell types between reference and query datasets. To evaluate the method's ability to detect novel cell types, we considered the identification of novel cell types as a binary classification problem. We reported the threshold-free AUROC based on the prediction confidence, where novel cell types were assigned a value of one and otherwise zero. As indicated by Dhamija et al. [19], we also report the Open-Set Classification Rate (OSCR), which measures the trade-off between accuracy and novel cell type detection rate as the confidence threshold varies. This metric provides a nuanced evaluation of the model’s ability to identify novel cell types while maintaining overall accuracy.

On the other hand, to more rigorously assess the interpretability of CellPredX. To this end, we have introduced a new quantitative metric, Key Gene Hit Rate (KGHR), to evaluate the biological relevance of the identified determinant features. For each predicted cell type, we first ranked determinant features for each cell according to their contribution scores and selected the top 20 features. We then compared these features with the top 10 differentially expressed genes (DEGs) identified for that cell type in the reference dataset. The KGHR was calculated as the proportion of overlapping genes between these two sets, defined as:

$$\begin{aligned} KGHR=\frac{\left| Top 10 determinant features\cap Top 10 DEGS \right|}{10}\#\left( 1 \right) \end{aligned}$$

A higher KGHR indicates greater consistency between the model’s inferred determinant features and biologically validated marker genes, thereby reflecting the interpretability and biological fidelity of the CellPredX framework.
